# Identifying lifestyle factors associated to co-morbidity of obesity and psychiatric disorders, a pilot study

**DOI:** 10.3389/fpubh.2023.1132994

**Published:** 2023-05-03

**Authors:** Christine Gaskell, Padmakumari Sarada, Eiman Aleem, Ghizlane Bendriss

**Affiliations:** ^1^Premedical Division, Weill Cornell Medicine, Ar-Rayyan, Qatar; ^2^Biomedical Science, London South Bank University, London, United Kingdom

**Keywords:** obesity, overweight, Qatar, UK, nutrition, depression

## Abstract

Obesity and psychiatric disorders are linked through a bidirectional association. Obesity rates have tripled globally in the past decades, and it is predicted that by 2025, one billion people will be affected by obesity, often with a co-morbidity such as depression. While this co-morbidity seems to be a global health issue, lifestyle factors associated to it differ between countries and are often attributed to more than one factor. Prior obesity studies were performed in Western populations; this is the first study that investigates lifestyle factors relating to obesity and mental health of the diverse population in Qatar, a country that has witnessed tremendous lifestyle change in a short time. In this pilot study, we surveyed 379 respondents to assess and compare the lifestyles of Qatar residents to the global population. However due to the high proportion of responses from the United Kingdom (UK) residents, we have made comparisons between Qatar residents and UK residents. We used chi-square analysis, spearman rank correlation and logistic regression to compare the lifestyle factors of individuals suffering from both increased BMI and mental health conditions. The types of food consumed, stress, exercise frequency and duration, alcohol and tobacco consumption, and sleep duration, were explored and results argue that different lifestyle factors can contribute to the same health condition, suggesting different mechanisms involved. We found that both groups reported similar sleep durations (*p* = 0.800), but that perception of sleep (*p* = 0.011), consumption of alcohol (*p* = 0.001), consumption of takeaway food (*p* = 0.007), and physical activity significantly varied between the groups (*p* = 0.0001). The study examined the predictors of comorbidity in Qatar as well as UK populations using multivariate logistic regression analysis. The result of the study showed no statistical association between comorbidity and the predictors drinking habit, smoking, physical activity, vegetable consumption, eat outs, and sleep perception for the Qatar population, and for the combined population. This study, however showed a significant association (*p* = 0.033) between sleep perception and comorbidity for the UK population. We conclude that further analysis is needed to understand the relationship between specific lifestyle factors and multimorbidity in each country.

## 1. Introduction

Over the last three decades, Qatar has witnessed an increase in citizens that are either overweight or obese (1). Simultaneously, anxiety and depressive disorders are the most common psychiatric disorders in Qatar, with prevalence being comparable to the rest of the world (2).

Many co-morbidities have been associated with practicing an unhealthy lifestyle, including obesity, psychiatric disorders, diabetes type 2, an increased risk of developing certain cancers, osteoarthritis, and cardiovascular disease (3–7). Notably, psychiatric disorders have been associated with having a BMI greater than 25 (8). Individuals with low self-esteem and distorted body image suffer from poor mental health, and those with poor mental health have displayed a tendency to over-eat. Both of these factors, overeating and psychiatric disorders impact an individual’s quality of life and morbidity (9).

While the co-morbidity between psychiatric disorders and BMI greater than 25 has been observed in various countries, we inquire on whether the lifestyle factors associated to it are the same. Is there a “one-size-fits-all” approach to targeting this co-morbidity in terms of behavioral changes? The following pilot study aims at exploring this question by comparing the lifestyle between Qatar and a western country.

Originally, the survey was distributed through social media, and the country from which we received most answers beside Qatar was UK. This is not surprising as around 22,000 UK citizens are currently residing in Qatar, whilst Qatar citizens and businesses have over £40 billion in investments in the UK, and around 100,000 Qataris regularly visit the UK for travel and education (10). Despite differences such as climate, population, and culture, the two countries are connected on multiple levels.

To shed more light on the role of each lifestyle factor and their interactions, this paper looks to analyze the cultural and environmental lifestyle factors of those with a psychiatric disorder and BMI greater than 25 residing in Qatar and compare it to those residing in the UK to see if there are any similarities or differences in their lifestyles.

Qatar is a small peninsula located in the Persian Gulf. It has a population of approximately 2.6 million people as of 2021 (11), while the UK has a much larger population than Qatar at over 67 million in 2020 (12). Life expectancy in both Qatar and the UK is 79 years old for males and 82 and 83 years old, respectively, for females. Both countries despite the geographical distance have very similar national dishes, both being based around rice and spices. A consensus by Cloud Based Human Resource Software (CIPHR) found that 39% of people resident in the UK list money worries as a main stressor, whilst a study from Qatar concluded that work pressure was the root cause of stress in Qatar (13, 14).

Food is an important part of Qatari and Arabic culture, with countries in the Gulf region known for their hospitality. Qatar has also become a melting pot of culture whereby it is easy to access a variety of cuisines, contributing to Qatar residents consuming more animal proteins, fat, and refined carbohydrates. Currently, it is difficult to compare nutrition in Qatar vs. UK because most studies completed in Qatar focus specifically on Qatar nationals and not Qatar residents, which does not represent a true statistic since the large expatriate population is thus excluded. A previous study has shown that 70.1% of Qatar citizens had a BMI over 25 (15). Another study investigated rates of overweight and obesity in school-age children who were both citizens and residents and found that Qatari nationals were 1.4 times more likely to be obese than non-Qataris (16). Indeed, the types and volume of food consumed between the two groups has been showed to be different, Qatari individuals consuming around 4,275 kcal daily, substantially more than non-Qatari households, which consume around 2,424 Kcal daily (17). Dining out and purchasing takeaway food is common in Qatar, unlike in the UK (15). In comparison, the average daily intake in the UK is still lower at 1764 Kcal, with 56.8% of the calories coming from ultra-processed foods (18).

The consistently high temperatures in Qatar, which average 45 degrees Celsius and humidity of up to 94% in the summer months, constrain most outdoor activities and sports to the winter months. Thus, the temperature not only affects people’s ability to exercise but also their daily routines. During the summer months, it is common for people to wait until later in the evening to socialize, meaning that they go to bed later and potentially sleep less, as work and school hours tend to start relatively early in the morning. Whilst in the UK 38% of men and 23% of women regularly take part in aerobic exercise and strength training (19).

The combination of low physical activity levels, less sleep, and a diet high in processed foods has been shown to negatively impact individuals’ mental health (20). To gain insight into the local impact of a country’s culture on the lifestyle of its residents, this study aimed to compare the lifestyle and health of residents of Qatar and the UK. We designed an observational cross-sectional study that was conducted between August 2021 and March 2022 to find answer the following questions: How different is diet and nutrition in Qatar and UK? How different is alcohol consumption in Qatar and UK? How different is tobacco use in Qatar and UK? How different is sleep in Qatar and UK? How different is physical activity in Qatar and UK? What correlations between lifestyle factors, BMI and neuropsychiatric disorders can be revealed by this comparison?

## 2. Methods

### 2.1. Sample population

We designed a questionnaire for the general public according to the protocol approved by the institutional review board. The questionnaire was prepared using Qualtrics software in the English language, and links were generated for distribution via internal email and relied on social media sharing, via Instagram, Twitter, and Facebook. The questionnaire was open to participants globally who were over the age of 18 years old and were able to read English. In total, 384 responses were recorded from participants all over the world, most of which were collected during the first few weeks of the release (August 2021). Table 1 represents the number of respondents and their country of residence.

**Table 1:** Country and number of respondents to the questionnaire.

Country of residence	Number of respondents	Relative frequency %
Qatar	294	77.57%
UK	63	16.62%
France	5	1.31%
Saudi Arabia	3	0.79%
UAE	3	0.79%
Oman	2	0.52%
Egypt	2	0.52%
United States of America	2	0.52%
South Korea	1	0.26%
Bahrain	1	0.26%
Spain	1	0.26%
Russia	1	0.26%
Philippines	1	0.26%
Total	379	100%

For the sake of this present analysis, only respondents who filled both criteria of BMI > 25 and a psychiatric disorder have been considered for the comparison. Respondents having a BMI > 25 and a psychiatric disorder is an example of co-morbidity, which is a condition of having two or more diseases at the same time.

#### 2.1.1. Questionnaire

The lifestyle questionnaire included 40 questions on social demographic variables, exercise, sleep, diagnosed diseases, nutrition, stress and depression, alcohol and tobacco consumption. We constructed the questions so that evidence-based feedback was provided to the participants based upon their answers. The subsections below detail the questions and answers used for this paper. A table of all the questions asked and the scoring assigned to answers can be found in Supplementary File (S1). We also obtained approval from the American College of Lifestyle Medicine to feature ACLM’s flyers in between question blocks as a means to advocate for healthy lifestyle choices.

#### 2.1.2. Social demographic variables

The questions used to assess the social demographic variables for this data set were not scored and were collected in the following format: Height (Answer recorded in cm), Weight (Answer recorded in kg) Country of residence (Qatar, Other. If other is selected prompted to answer with country name)

#### 2.1.3. Exercise

A disclaimer was given at the start of the subset of questions: Medical disclaimer: This quiz does not provide medical advice. It is intended for informational purposes only. According to the American College of Sport Medicine guidelines and the American College of Lifestyle Medicine, if you have symptoms of metabolic disease, cardiovascular disease or renal disease, or if you have been diagnosed with one of these diseases, you must obtain medical clearance before engaging in any exercise. A single question was used from the data set to assess exercise between the two groups. Answers were scored and feedback given dependent on the participants answer. The exercise variable was collected in the following format: Days exercised in a week (0, 1–3, 4–6, 7). For the logistic regression analysis, we grouped the results into two groups, those who did 0 days exercise and those who 1 day or more.

#### 2.1.4. Sleep

Two questions from the data set were used for the sleep section. Sleep duration was scored, and feedback given dependent on the participants answer. Perception of enough sleep was not scored. Sleep variables were collected in the following format: Hours slept in an average night (Less than 4, 4–6 h, 7–9 h, 10+ hours), perception of having enough sleep (Yes, no). For the logistic regression analysis, we grouped the results of sleep duration into two groups, those who slept less than 4 h and those who slept 4 or more hours.

#### 2.1.5. Diseases

A single question was used to assess the diagnosed diseases for this data set and was not scored. The diagnosed disease variable was collected in the following format: Have you ever been diagnosed with any of the following medical conditions by a GP/physician? Diabetes type 1, Diabetes type 2, Cardiovascular disease, Anxiety, Obesity, Cancer (if selected prompted to name which type), Inflammatory bowel disease, Rheumatoid arthritis, Autoimmune disease, Autism, Depression, Schizophrenia, Bipolar, Alzheimer’s, Parkinson’s, Arthritis.

Two questions from the data set were used for self-reported stress and depression, both were scored, and feedback given dependent on the participants answer. The variables were collected in the following way: Depression scale- Over the last 2 weeks, how often have you been bothered by any of the following problems? 1. Little interest or pleasure in doing things, 2. Feeling down, depressed, or hopeless, 3. Trouble falling or staying asleep, or sleeping too much, 4. Feeling tired or having little energy, 5. Poor appetite or overeating, 6. Feeling bad about yourself or that you are a failure or have let yourself or your family down, 7. Trouble concentrating on things, such as reading the newspaper or watching television, 8. Moving or speaking so slowly that other people could have noticed. Or the opposite being so fidgety or restless that you have been moving around a lot more than usual, 9. Thoughts that you would be better off dead, or of hurting yourself (Not at all, Several days, More than half the days, Nearly every day). The depression scale used is from the American Psychological Association and total scores of 1–4 were classed as minimal depression and not counted. Perceived stress scale- In the last month, how often have you felt that you were unable to control important things in your life? In the last month, how often have you felt confident about your ability to handle your personal problems? In the last month, how often have you felt that things were going your way?

In the last month, how often have you felt difficulties were piling up so high that you could not overcome them? (Never, almost never, sometimes, fairly often, very often). The stress scale used is from the American College of Lifestyle Medicine and scored of 0–4 were classed as low stress and not counted.

#### 2.1.6. Nutrition

Three questions from the data set were used for the nutritional comparison, two were scored, and feedback given dependent on the participants answer. The nutrition variables were collected in the following format: Vegetables consumed daily (1, 2, 3, 4+), Takeaways/times dined out in a week (0,1,2,3, 4+), Food type most eaten 1 = most eaten, 8 = least eaten (red meat, poultry pasta, cheese, pizza/ sandwich/ hamburger, vegetable, pastries/sweets). For the logistic regression analysis, we grouped the results into two groups, for vegetable consumption those who ate less than 4 servings of vegetable and those who ate four or more servings of vegetables. For takeaways/dining out the results were grouped into those who dine out/ eat takeaway less than 3 times a week and those who dine out/ eat takeaway 3 or more times a week.

#### 2.1.7. Alcohol and tobacco consumption

Two questions from the data set were used for alcohol and tobacco consumption, both were scored, and feedback given dependent on the participants answer. The variables were collected in the following format: Consumption of alcohol (yes, no), use of nicotine products (yes, no).

### 2.2. BMI calculation

The formula used to calculate BMI is: (person’s weight in kilograms) divided by their squared height in meters.

### 2.3. Psychiatric disorder assessment

The Patient Health Questionnaire (PHQ-9) of the American Psychological Association was used to assess depression. The assessment includes DSM-IV depression criteria and other leading major depressive symptoms into a brief self-report set of nine questions commonly used for screening and diagnosis (21). The second scale used is the perceived stress scale- 4 (PSS-4) (22). For both scales, participants who scored 5 and above (at least mild depression) were counted as having a psychiatric disorder.

### 2.4. Statistical analysis

SPSS 26 software was used for descriptive and statistical analysis. The frequency and percentages for nominal variables were described. Pearson’s Chi-square test for categorical variables was used to analyze the lifestyle variables of the respondents from Qatar and UK. All Chi- square tests used were two sided at the level *α* = 0.05. A Spearman rank correlation was done to analyse the correlations between independent variables. Multivariate logistic regression analysis was used to determine if the independent variable has any effect on the dependent variable, co-morbidity. A second logistic regression test was conducted with country of residence entered as an independent variable. The results of the logistic regression analysis were presented as value of p, odds ratio (OR) and 95% confidence interval (95%CI). The case processing summary for Qatar (Supplementary Table 1) and the UK (Supplementary Table 2) and parameter estimates for Qatar (Supplementary Table 12) and the UK (Supplementary Table 13) containing the descriptive data output from the logistic regression analysis can be found in the supplementary file to reduce the number of tables in the body if the paper.

## 3. Results

### 3.1. Co-morbidity assessment

**Figure 1:**
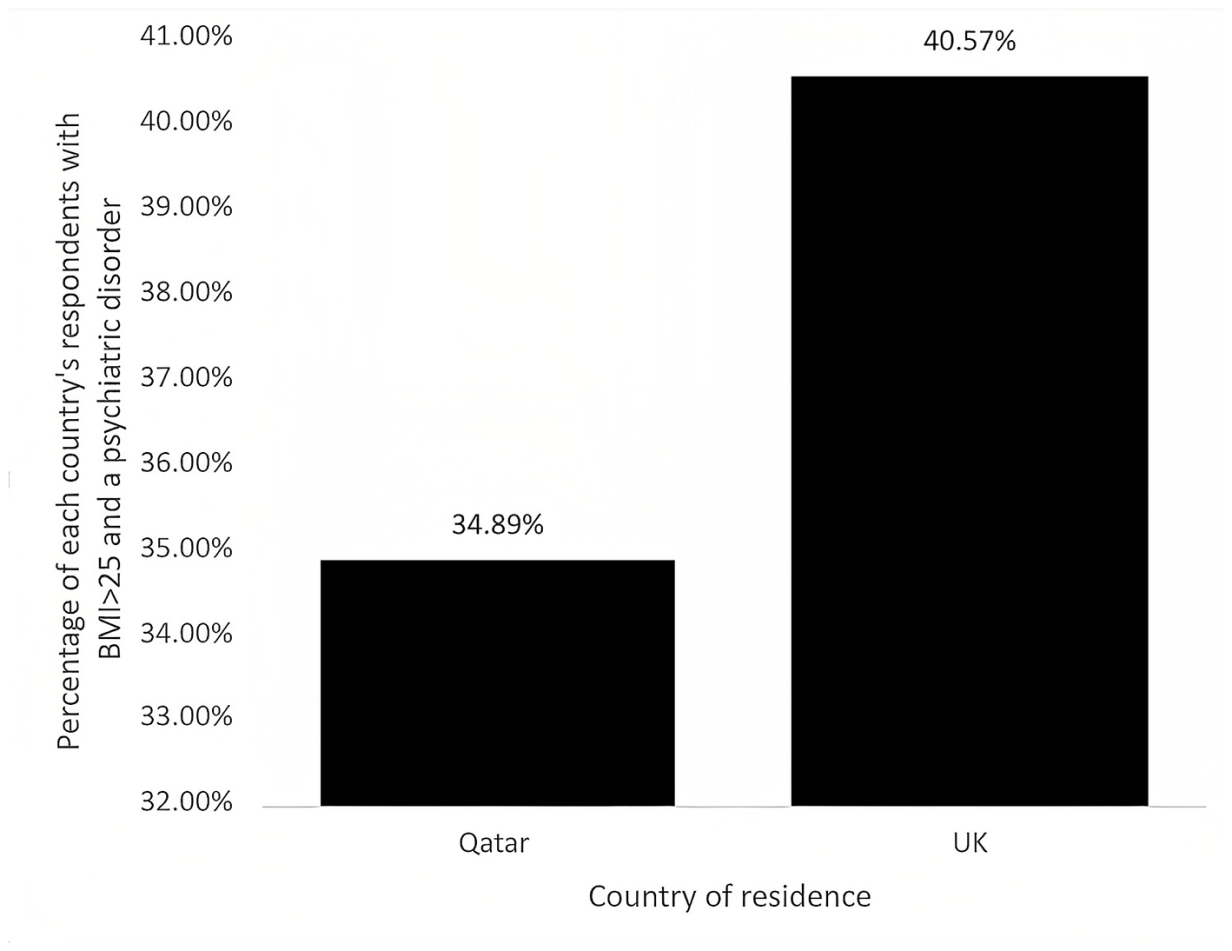
Co-morbidity of psychiatric disorder and BMI > 25 in Qatar’s and UK’s respondents. Percentages correspond to the percentage of respondents from respective country who had both a BMI > 25 and a psychiatric disorder.

Of the respondents to the questionnaire, 294 were resident in Qatar and 63 were resident in the UK. Of those respondents, 104 (35.37%) from Qatar and 28 (40.57%) from the UK had the co-morbidities of a BMI >25 and psychiatric disorder. The percentages of respondents from Qatar and UK with the co-morbidity are displayed in Figure 1. The respondents were then separated into groups dependent on country of residence, the number of diagnosed morbidities (Table 2) and types of diseases (Table 3). The condition most frequently reported by participants was “Psychiatric disorders” (Table 3). In total, 22 respondents from Qatar and 11 from the UK were diagnosed by a doctor as having a psychiatric disorder (Table 4), the remaining 99 did not report it but were scored with at least a mild depression or mild perceived stress using our validated scales. A chi-square test was used to assess if there was a statistical difference between those diagnosed by a doctor and was found to be significantly different between countries with a *p*-value = 0.013. Eleven respondents from Qatar and zero respondents from the UK were diagnosed as being obese by a doctor (Table 4), the remaining 121 were concluded by the calculation of reported height and weight as per the formula described in the methods section.

**Table 2:** Number of morbidities diagnosed by a doctor in all the respondents.

Number of morbidities	Number of respondents^a^	Relative frequency %
1 disease	86	66.66%
2+ diseases	43	33.33%
Total	129	100%

a Number of respondents who answered this question in the questionnaire, *n* = 129.

**Table 3:** Morbidities diagnosed by a doctor in all respondents.

Diagnosed morbidity type	Number of respondents^a^	Relative frequency %
Psychiatric disorders	92	53.17%
Obesity	25	14.45%
Autoimmune	19	8.09%
Type 2 diabetes	11	6.35%
IBD	7	4.04%
Arthritis	7	4.04%
Cardiovascular disease	5	2.89%
Rheumatoid arthritis	4	2.31%
Type 1 diabetes	3	1.73%
Total	173	100%

a Number of respondents who answered this question in the questionnaire, *n* = 173.

**Table 4:** Respondents with the co-morbidity (BMI > 25 + psychiatric disorder) and their diagnosis status.

		Qatar	UK	Chi-square	*p*-value
Psychiatric disorder diagnosed by scale scoring	Yes	82 (82.8%)	212 (82.2%)	0.021	0.884
	No	17 (17.2%)	46 (17.8%)		
Psychiatric disorder diagnosed by physician	Yes	22 (66.7%)	272 (84%)	6.156	0.013
	No	11 (33.3%)	52 (16%)		
BMI25 > diagnosed by a physician	Yes	12 (100%)	282 (81.7%)	2.661	0.136
	No	0 (0%)	63 (18.3%)		
BMI25 > diagnosed by BMI calculator	Yes	92 (76.7%)	202 (85.2%)	4.022	0.045
	No	28 (23.3%)	35 (14.8)		

### 3.2. Sleep assessment

The number of responses received for the sleep assessment questions were n = 28 for the UK, while Qatar had *n* = 102 responses for hours slept and *n* = 103 responses for perception of having enough sleep (Table 5). A chi-square test was used to assess average hours slept per night by country of residence. The chi-square test was not statistically significant, 𝑥^2^ (1, *N* = 130). =0.924, *p*-value = 0.670.

**Table 5:** A comparative analysis of lifestyle factors between Qatar and UK residents.

	Qatar	UK	Chi-square	*p*-value
Hours slept				
<4	3 (2.9%)	1 (3.6%)	0.924	0.800
4–6	43 (42.2%)	14 (50%)		
7+	56 (54.9%)	13 (46.4%)		
Perception of having enough sleep				
Yes	54 (52.4%)	7 (25%)	6.656	0.011
No	49 (47.6%)	21 (75%)		
Alcohol consumption				
Yes	23 (22.3%)	15 (53.6%)	10.434	0.001
No	80 (77.7%)	13 (46.4)		
Tobacco consumption				
Yes	14 (13.6%)	1 (6.7%)	.	0.689
No	89 (86.4%)	14 (93.3%)		
Takeaways/ dining out in a week				
0	10 (9.6%)	5 (17.9%)	13.353	0.007
1	22 (21.2%)	14 (50%)		
2	31 (29.8%)	6 (21.4%)		
3	28 (26.9%)	2 (7.1%)		
4+	13 (12.5%)	1 (3.6%)		
Daily vegetable serving				
0	8 (7.5%)	1 (3.6%)	8.604	0.058
1	42 (40.38%)	7 (25%)		
2	36 (34%)	8 (28.6%)		
3	11 (10.4%)	6 (21.4%)		
4+	7 (6.6%)	6 (21.4%)		
Days of exercise per week				
0	31 (30.1%)	2 (7.1%)	20.409	0.0001
1–3	43 (41.7%)	5 (17.9%)		
4–6	26 (25.2%)	20 (71.4%)		
7	3 (2.9%)	1 (3.6%)		

Among the UK residents, 25% had the perception they had enough sleep, whilst 75% felt they did not get enough sleep in an average night. For Qatar residents, 52.4% felt they had enough sleep, and 47.6% felt they did not get enough sleep in an average night. A chi-square test was used to assess whether country of residence was related to the perception of having enough sleep. The Pearson chi-square test was statistically significant, 𝑥^2^ (1, *N* = 131) =6.656, *p*-value = 0.011, with Phi (𝜑) coefficient of −0.225, indicating a small relationship.

### 3.3. Nutrition and exercise assessment

The number of UK respondents who answered the nutrition and exercise assessment questions was *n* = 28, and for Qatar *n* = 104 for take away and dine out and for daily vegetable consumption, and *n* = 103 for days of exercise per week (Table 5). A chi-square test was used to assess whether weekly take away/ dining out consumption was related to country of residence. The chi-square test was statistically significant, 𝑥^2^ (1, *N* = 132) =13.353, *p*-value = 0.007, with Phi (𝜑) coefficient of 0.326, indicating a small to medium relationship.

The percentage of Qatar residents who consume less than the recommended two portions of vegetables per day is 49%, while the percentage of UK residents is 28.6%. A chi-square test was used to assess whether daily vegetable consumption was related to country of residence. The chi-square test was not statistically significant, 𝑥^2^ (1, *N* = 134) =8.604, *p*-value = 0.058.

The top five most eaten foods in Qatar were poultry, vegetables, rice, red meat, and pasta (Supplementary Table 14). This information was collected as part of the questionnaire; however, respondents were not asked how these foods were cooked and consumed (i.e., fried, baked, part of a dish, as an individual item, etc.).

The percentage of respondents with the co-morbidity who exercised 0 times a week was 30.1% for Qatar vs. 7.1% for UK. A chi-square test was used to assess whether days of exercise per week was related to country of residence. The chi-square test was statistically significant, 𝑥^2^ (1, *N* = 131) =20.409, *p*-value = 0.0001, with Phi (𝜑) coefficient of 0.405, indicating a small to medium relationship between the country of residence and the number of days exercising per week.

### 3.4. Risky substances

The number of respondents with the co-morbidity from UK who provided data on risky substances was *n* = 28 and Qatar *n* = 103 (Table 5). Meanwhile, 22.3%. of Qatar respondents reported that they consume alcohol, vs. 53.6% of UK respondents. A chi-square test was used to assess whether alcohol consumption was related to country of residence. The chi-square test was statistically significant, 𝑥^2^ (1, *N* = 131) =10.434, *p*-value = 0.001, with Phi (𝜑) coefficient of 0.282, indicating a small to medium relationship between the country of residence and alcohol consumption.

The percentage of Qatar respondents with the co-morbidity that use tobacco products was 13.6%. The percentage of UK residents that use tobacco products was 6.7%. A chi-square test was used to assess whether tobacco use was related to country of residence. The chi-square test was not statistically significant, 𝑥^2^ (1, *N* = 118), *p*-value = 0.689.

### 3.5. Logistic regression

Spearman’s correlation test (Table 6) was conducted to see the correlation between the predictor variables as an assumption check for the logistic regression and found no significant correlation between the independent variables (None of the correlation coefficients are more than 0.6 or less than −06).A multivariate logistic regression model (Table 7) analyzed the effect of drinking habit (alcohol consumption), smoking (use of nicotine or tobacco products), physical activity (do you exercise regularly?), vegetable consumption (daily vegetable servings), eat outs (dine outs or take aways per week), hours slept per night and perception of having enough sleep on comorbidity in Qatar population (*N* = 294) and UK (*N* = 63) separately, and also for the combined population. The logistic regression model showed no statistical significance when all the predictor variables were considered and comorbidity for Qatar population and the population as a whole, however for UK population, significance at *p* < 0.05 level demonstrated a statistical association for sleep perception (do you feel you are getting enough sleep?) and comorbidity, OR. 3.415 (95% CI = 1.105, 10.552), *p* = 0.033 (Table 8).

**Table 6:** Spearman’s rank correlations between the predictor variables.

Spearman’s rho correlations									
		Alcohol consumption	Tobacco consumption	Sleep perception	Dine outs/ takeaways	Daily Vegetable	Exercise frequency	Country	Sleep hours
Alcohol consumption	Correlation Coefficient	1.000	0.099	0.002	−0.108^*^	−0.102	−0.185^**^	0.295^**^	−0.091
	Sig. (2-tailed)	.	0.074	0.977	0.050	0.063	0.001	0.000	0.100
	*N*	329	329	329	329	329	329	329	329
Tobacco consumption	Correlation Coefficient	0.099	1.000	−0.069	0.068	0.001	−0.015	−0.099	−0.046
	Sig. (2-tailed)	0.074	.	0.210	0.221	0.986	0.780	0.072	0.403
	*N*	329	329	329	329	329	329	329	329
Sleep perception	Correlation Coefficient	0.002	−0.069	1.000	−0.044	0.038	0.131^*^	0.106	0.133^*^
	Sig. (2-tailed)	0.977	0.210	.	0.423	0.489	0.018	0.054	0.016
	*N*	329	329	329	329	329	329	329	329
Dine outs/takeaways	Correlation Coefficient	−0.108^*^	0.068	−0.044	1.000	0.127^*^	0.222^**^	−0.208^**^	−0.038
	Sig. (2-tailed)	0.050	0.221	0.423	.	0.020	0.000	0.000	0.498
	*N*	329	329	329	338	338	332	338	329
Daily Vegetable serving	Correlation Coefficient	−0.0102	0.001	0.038	0.127^*^	1.000	0.110^*^	−0.185^**^	−0.0091
	Sig. (2-tailed)	0.063	0.986	0.489	0.020	.	0.046	0.001	0.101
	*N*	329	329	329	338	338	332	338	329
Exercise frequency	Correlation Coefficient	−0.185^**^	−0.015	0.131^*^	0.222^**^	0.110^*^	1.000	−0.170^**^	−0.019
	Sig. (2-tailed)	0.001	0.780	0.018	0.000	0.046	.	0.002	0.731
	*N*	329	329	329	332	332	332	332	329
Country	Correlation Coefficient	0.295^**^	−0.099	0.106	−0.208^**^	−0.185^**^	−0.170^**^	1.000	−0.013
	Sig. (2-tailed)	0.000	0.072	0.054	0.000	0.001	0.002	.	0.808
	*N*	329	329	329	338	338	332	357	329
Sleep hours	Correlation Coefficient	−0.091	−0.046	0.133^*^	−0.038	−0.091	−0.019	−0.013	1.000
	Sig. (2-tailed)	0.100	0.403	0.016	0.498	0.101	0.731	0.808	.
	*N*	329	329	329	329	329	329	329	329

* Correlation is significant at the 0.05 level (2-tailed).

** Correlation is significant at the 0.01 level (2-tailed).

**Table 7:** Multivariate sesults for the combined sample Qatar and UK.

Characteristics	*p*-value	OR (95% CI)
Alcohol consumption (ref: No)	0.164	0.688 (0.406, 1.165)
Tobacco consumption (ref: No)	0.669	1.176 (0.559, 2.475)
Sleep perception (ref: Yes, to the question, do you feel you get enough sleep?)	0.889	1.033 (0.655, 1.630)
Dine out/take away (ref: <3 per week)	0.202	1.395 (0.837, 2.326)
Daily Vegetable Servings (ref: ≥4 servings daily)	0.433	1.340 (0.645, 2.787)
Exercise frequency (ref: Yes)	0.424	1.256 (0.718, 2.196)
Country (ref: Qatar)	0.088	1.691 (0.924, 3.093)

**Table 8:** Multivariate logistic regression results for each country separately.

Characteristics	*p*-value	OR (95% CI)
Qatar		
Alcohol consumption (ref: No)	0.344	0.739 (0.394, 1.384)
Tobacco consumption (ref: No)	0.604	1.232 (0.560, 2.708)
Sleep perception (ref: Yes, to the question, do you feel you get enough sleep?)	0.236	0.728 (0.431, 1.231)
Dine out/take away (ref: <3 per week)	0.289	1.341 (0.779, 2.308)
Daily Vegetable serving (ref: ≥4 servings daily)	0.487	1.401 (0.541, 3.624)
Exercise frequency (ref: Yes)	0.252	1.417 (0.781, 2.571)
UK		
Alcohol consumption (ref: No)	0.203	0.494 (0.167, 1.461)
Tobacco consumption (ref: No)	0.987	1.021 (0.074, 14.071)
Sleep perception (ref: Yes, to the question, do you feel you get enough sleep?)	0.033	3.415 (1.105, 10.552)
Dine out/take away (ref: <3 per week)	0.807	1.237 (0.224, 6.832)
Daily Vegetable serving (ref: ≥4 servings daily)	0.436	1.668 (0.460, 6.044)
Exercise frequency (ref: Yes)	0.891	(0.131, 5.842)

## 4. Discussion

### 4.1. A silent co-morbidity: the problem of awareness

An important proportion of respondents from Qatar (89.42%) was found with BMI greater than 25. Our survey noted that only 25% of those who had a BMI greater than 25 and a psychiatric disorder had been diagnosed by a doctor. The remaining 75% self-identified via the questionnaire. This has been previously highlighted in a study that found that only 6.7% of men and 22.2% of woman correctly identified themselves as being obese (23).

Having a BMI greater than 25 has been linked to co-morbidities, such as metabolic syndrome, type 2 diabetes, and cardiovascular disease (24) which are now the leading causes of deaths in the world (25). In this study, we have observed that at least half of those with psychiatric disorders were also suffering from increased BMI, both in Qatar and UK. Interestingly, the majority of respondents were also undiagnosed in their psychiatric disorders, with 79.8% of Qatar respondents and 60.7% of UK respondents being classified as having a psychiatric disorder based on the answers given in the survey as opposed to being diagnosed by a doctor. This is far higher than a previous study conducted in 2014, which noted that 36% of common psychiatric disorders in the UK are undiagnosed (26).

This pilot study suggests that for larger cohorts labelled as “healthy” as the ones provided by Qatar Biobank, it will be important to look for the existence of this co-morbidity. This also highlights the importance of raising awareness on both conditions, and help them move out of the “pre-contemplation” stage of the transtheoretical model of behavior change (27). Indeed, if a person is unaware that they have a health condition such as a BMI greater than 25 or a psychiatric disorder, they are unable to make the changes needed to improve their health and lifestyle factors associated to their condition.

### 4.2. Lifestyle factor 1: exercise

According to the questionnaire results, around 30.1% of Qatar’s respondents with the co-morbidity did no weekly exercise, with only 25.6% exercising 4–6 times per week. This is not surprising since the weather condition in the desertic climate of Qatar is relatively hot all year long and might be discouraging people to exercise outdoor. In the past few years, Qatar has launched several initiatives to promote and facilitate outdoor physical activities such as public parks, the National Sport Day, health campaigns such as the “Step into health.” It would be interesting to observe this specific lifestyle factor over the coming years on a larger cohort.

In comparison, 71.4% of UK residents with the co-morbidity exercised 4–6 times a week, with only 7.1% reporting that they did not partake in any weekly exercise. This suggest that exercise might play a heavier role in the co-morbidity found in Qatar than in UK and might therefore be the preferred lifestyle factor to target in Qatar for managing the co-morbidity, but not in UK.

Using exercise as an alternative to medication for treating psychiatric disorders has only recently been investigated, but findings indicate that exercise can have a positive effect on an individual’s mood through the increase in endorphins and a decrease in cortisol (28). Exercise has also been shown to stimulate the growth of new nerve cells and the release of proteins such as brain-derived neurotrophic factor, which is essential to growing and maintaining neurons involved in emotion, as well as increasing the size of the hippocampus and enhancing cognitive function (29–32). Studies have shown that observing the recommended guidelines of 150 min moderate or 75 min vigorous physical activity/exercise is not enough to promote significant weight loss (33, 34). Instead, it was suggested that 200–300 min per week of exercise is an optimal amount of physical activity to accomplish significant weight loss (35). This recommendation, however, does not take into account training methods such as resistance training or weight training, which are often effective in reducing fat mass and the associated negative health implications even if no weight loss is observed (36). Nonetheless, it is recognized that exercise alone cannot solely contribute to weight loss, especially when diet is not healthy and balanced.

### 4.3. Lifestyle factor 2: nutrition

Between 2009–2015, Qatari children between the ages of 12–17 had the highest levels of fast-food consumption in the region, on average consuming fast food over 2.5 times per week (37). This correlates to the findings from the questionnaire, which show that 69.2% of Qatar residents with the co-morbidity dine out or consume take away food 2 or more times per week. This is doubled in comparison to the 32.1% of UK residents with the co-morbidity who dined out or consumed takeaway food 2 or more times per week. This suggests that nutrition is a second lifestyle factor that can be preferably considered as a target for change.

Stress-induced overeating leads to obesity, which has a direct impact on neurotransmitters and inflammatory markers that are present and affect mood, with a high-fat diet thought to cause mood disorders (38). On the other hand, psychiatric disorders are known to cause over-eating and binge-eating and curtail participation in exercise, which leads to increased levels of body fat (39). We are here in the presence of a bidirectional communication between gut and brain, commonly called as the gut-brain axis (40). This is supported by a study that found that eating fruit and vegetables containing dietary fiber was associated with better mental health (41). A review that evaluated 61 observational studies asserted that adults who had a higher consumption of fruit, vegetables, and dietary fiber were protected against depressive symptoms (42). It has long been known that vegetables contain many of the antioxidants, vitamins and dietary fiber that our bodies need (43). Low grade inflammation has been found in individuals who are obese and overweight, this has been linked to causing metabolic changes and an accumulation of adipose tissue which plant peptides have been shown to have an impact on reducing (44). As well as the reduction of inflammatory cytokines, vegetables have been shown to be protective against cardiovascular disease, colon and rectal cancers and depression (44–46).

An increase in obesity levels is correlated with the increased consumption of a Western style diet, which tends to have more omega-6 and processed carbohydrates (47). For those with a BMI >25, there is a 44% increase in the risk of myocardial infarction, hypertension, fatty liver disease, type 2 diabetes, and some cancers (38, 48). Our results showed that in both Qatar and UK, the top consumed food was poultry, not vegetables. A study has shown that consuming a high amount of protein can enhance body composition and help to reduce body weight (49). However, many studies have proven that a diet predominantly composed of meat has a higher risk of diabetes, heart disease and stroke (50–52).

Nutrition itself affects lifestyle behavior and can influence of other lifestyle factors such as sleep.

### 4.4. Lifestyle factor 3: sleep

Nutrition affects sleep. Indeed, consumption of foods with a high glycemic index approximately 4 hours before bedtime increases REM sleep and reduces the onset of sleep latency (53, 54). However, a different study showed that individuals who consumed high fat and carbohydrate foods before bedtime had an increased sleep latency and decreased REM (55). Those with shortened sleep have shown to have a higher snack intake in the day, diets high in carbohydrates have been shown to cause an increase in REM sleep but the types of carbohydrates consumed cause different outcomes (56). This could be an explanation as to why only 52.4% of Qatar residents who responded felt that they had enough sleep in an average night.

Insufficient sleep has many negative effects on an individual’s health, including the development of many non-communicable diseases due to impairment of immune system and cardiovascular health as well as being linked to the development and worsening of psychiatric disorders (57).

Alcohol consumed before bedtime has been found to cause a decrease in the REM in the first half of the night, as the alcohol levels drop in the second half of the night sleep becomes disrupted at the time when REM duration is at its greatest causing an increase in waking leading to fatigue during the day (58). In our survey, a larger proportion of the UK residents (75%) felt that they did not get enough sleep. It is possible that this could be linked to alcohol consumption, with 53.6% of UK respondents confirming that they consume alcohol. Many people turn to alcohol to facilitate the sleep process, which is counterproductive, as alcohol causes an individual to have faster sleep onset but a poorer quality of sleep, with REM sleep being suppressed (59). This is due to the effects that alcohol has on many of the neurotransmitters, such as the GABAergic system, which is involved in sleep–wake regulation (59). Alcohol consumption can also disrupt sleep by disturbing the respiratory system (60). Further, alcohol consumption affects physical activity and exercise by decreasing strength output through inhibiting certain Ca2+ channels, decreasing muscle synthesis, peripheral vasodilation, and diuretic actions (61–63). In addition, the link between obesity and alcohol was highlighted back in 2014, when it was found that those who consumed alcohol had a 70% risk of obesity via the development of alcoholic fatty liver (64). Even though most obese individuals in Qatar do not drink alcohol, the metabolic syndrome is believed to arise from a combination of all other lifestyle factors, especially nutrition, sleep, and lack of physical activity.

### 4.5. Lifestyle factor 4: tobacco

Our study did not find any statistical difference in tobacco use between Qatar and UK respondents, but a significant difference in alcohol consumption. Tobacco and alcohol consumptions have been shown to be involved in with weight gain, by acting on sleep and modulated by the level of physical activity (64). It was found that cigarette smokers have a poorer sleep quality possibly due to nicotine being a stimulant; however, sleep quality was improved with a daily increase in exercise (65, 66). Physical exercise was found to deter adolescent girls from using tobacco products; however, the same result was not seen in adolescent boys (67).

The case of tobacco use is interesting as it relates to anxiety and weight gain at the same time, and its use is involved in the top causes for morbidity and mortality in the Western world (65). Our results show that a higher percentage of respondents from Qatar used tobacco products (13.6%) as compared to UK (6.7%). There is some conflict as to whether there is an association between people with a BMI >25 and people who smoke. Some people, especially females, believe that smoking will help in preventing weight gain (66). However, we are wondering whether respondents considered “shisha” as part of tobacco use, since the use of “shisha” is culturally more acceptable for women in the region. The amount of tobacco consumed were suggested to have an impact on the weight of an individual, with smokers less likely to be obese than non-smokers but only up to a certain level, heavy smokers are more likely to be obese than those who had never smoked (67). Research has also shown that people who quit smoking gain between 2.6 to 5.kg of weight which could be a decisive factor for people when considering stopping the use of tobacco products (68). An association was found between people with psychiatric disorders, heavy smoking, and difficulties in cessation of smoking (69). A barrier to stopping smoking for those with psychiatric disorders is the perception that it may worsen their symptoms due to many using smoking as a coping mechanism (70).

Other studies have suggested a strong link between alcohol and tobacco use, with up to 86% of smokers drinking alcohol, caused by environmental cues and genes that are involved in regulating some brain chemical systems such as cross tolerance; however those studies certainly did not account for countries such as Qatar were alcohol consumption is reduced (71, 72).

### 4.6. Correlation study: toward a paradigm shift

Surprisingly, the logistic regression analysis between lifestyle factors for UK, only found one factor to significantly correlate with the comorbidity: the question “Do you feel you get enough sleep?.” This question relates to quality of sleep rather than the number of hours slept. None of the other factor such as nutrition, exercise or alcohol consumption significantly correlated to the comorbidity. The small sample size for this group is an evident limitation, and the investigation need to be done for a larger group. However, when looking at the Qatar group, which had a bigger sample size, none of the lifestyle factors significantly correlated with the comorbidity, suggesting that in addition to the sample size factor, another reason might explain this observation. Our hypothesis is that two main factors might have contributed to this. The first one is that Qatar’s population is high in expatriates and includes many different cultural practices such as Indian, Asian, African, Arab, American, European, Eastern, which could result in large variations in answers. For example: eating three servings of vegetables a day might be critical for a group that does not include enough physical activity in their daily lifestyle but might be less critical for another group which exercises daily. Similarly, when the logistic regression analysis was conducted including both Qatar and UK participants, by including the country as an independent variable, the significance found for sleep perception was also lost. This might be due to large variations in lifestyles and the rather small sample size.

The second reason that could explain the failure to identify one of those lifestyle factors as significantly correlating to the co-morbidity may be that the lifestyle factors investigated in this study might be confounding factors to a different factor that was not investigated. In the light of previous studies on the gut-brain axis (73–76), we believe that this factor is the gut dysbiosis. Indeed, a growing body of evidence has shown that gut dysbiosis, or the sustained imbalance of gut microbes, is associated to nutrition (77) exercise (78), alcohol consumption (79) smoking (79), and sleep (80). In addition, gut dysbiosis has been association to obesity (81) and mental health (82). Indeed, gut dysbiosis has also been linked to mental health issues. A systematic review published in the journal Nutrients found that gut dysbiosis was associated with depressive symptoms in humans (83). In another study, mice with gut dysbiosis exhibited anxiety-like behaviors (84). These studies suggest that gut dysbiosis can have significant effects on mental health.

Emerging evidence suggests that an unhealthy gut microbiome can contribute to sleep disturbances, which can in turn lead to metabolic and mental disorders. Several studies have shown that gut dysbiosis can disrupt circadian rhythms and reduce the production of melatonin, leading to poor sleep quality and duration (85, 86). In a study of Leone et al. (87), mice fed a high-fat diet exhibited gut dysbiosis, which led to altered sleep patterns, increased food intake, and weight gain. These findings suggest that gut dysbiosis can result in sleep disturbances that contribute to metabolic dysfunction resulting in weight gain. Nevertheless, the gut dysbiosis can be caused by other factors such as nutrition, antibiotic courses history, substance use (88). A study published in the journal Frontiers in Psychiatry found that probiotics may improve sleep quality and reduce depressive symptoms in humans (89). These studies suggest that promoting gut health through dietary interventions may help improve sleep and reduce the risk of developing metabolic and mental disorders (90, 91). Studies on the role of the gut microbiome and metabolome in health and diseases have proposed a paradigm shift in health sciences (20, 78, 80, 81, 92). While our pilot study was only looking at lifestyle variables, the results suggest that the relationship of each variable to the comorbidity is complex and suggests that one or more pieces of the puzzle are missing. The development of a tailored strategies for the prevention and treatment of obesity and psychiatric disorders in Qatar and UK is needed in order to help reduce pressure on health services, ensure better quality of life and lower associated mortality rates.

## 5. Limitations

The data collected from the questionnaire include its reliance on individuals self-reporting, which can sometimes not be completely accurate. By making the questionnaire anonymous, we hoped that people would feel they could answer openly, this might have however helped in assessing depression and perceived stress disorders. With regards to using BMI as a measure for the different categories, we are aware that it is imperfect as it does not consider muscle mass, which can cause an individual to be classed as overweight or obese whilst they are in fact a healthy weight. Another issue with using BMI is that there are variations for different ethnic groups. The UK and Qatar groups were not equal in number, and the results of this study need to be confirmed by further explorations. We acknowledge the sample might not be representative of those who do not have access to Instagram, Facebook, twitter, WhatsApp or emails. As well as being limited by the method of data collection used.

The results cannot be generalized to the entire Qatar and UK population and this study is only a pilot study that aimed at providing preliminary data for further explorations. It was the first of its kind performed in the country, and the first to use this type of interactive survey. Testing the method was also an objective of this pilot study and we concluded that the survey was effective in recruiting a significant sample since 282 participants were recruited on the first week after the release of the advertisement on social media. Further exploration on larger cohort could get more insight by comparing the comorbid vs. total population.

## 6. Conclusion

**Figure 2:**
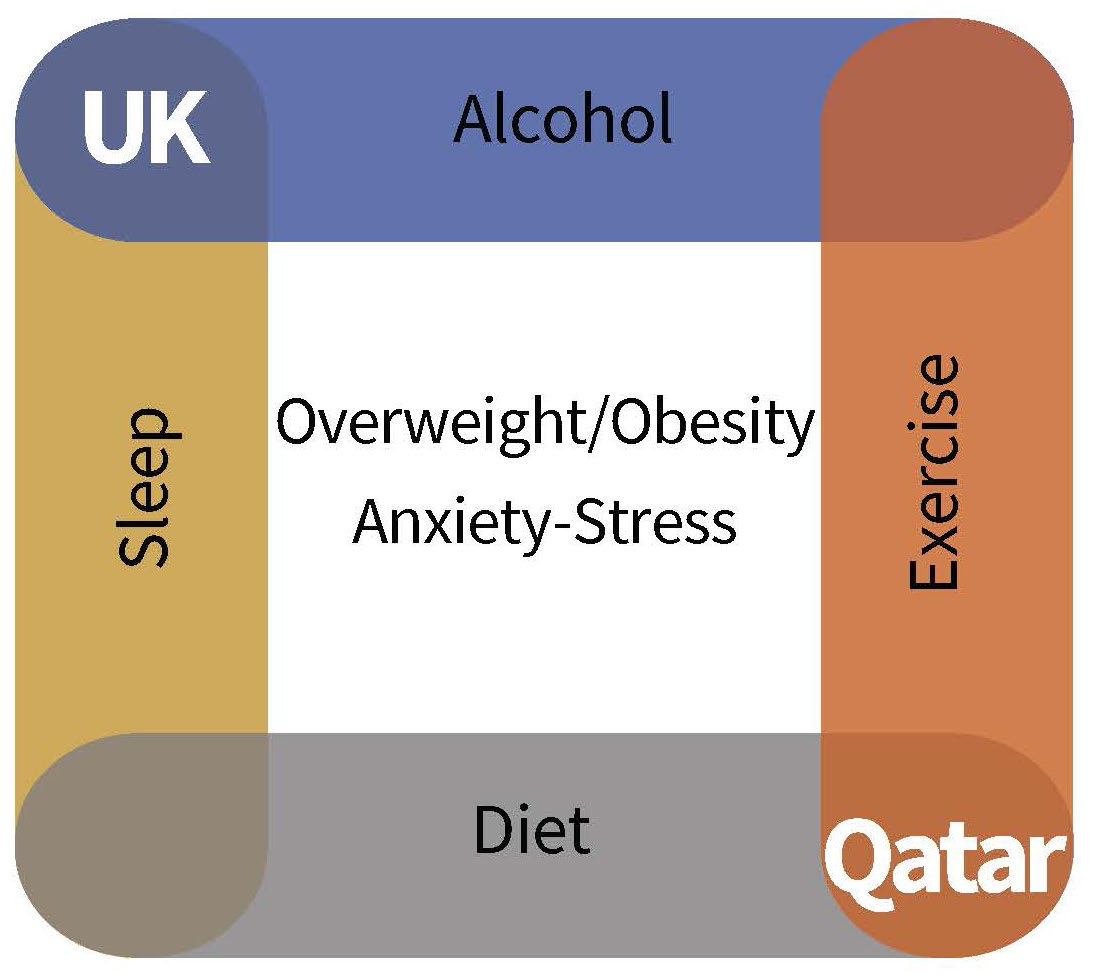
The need for precision medicine in lifestyle medicine.

The co-morbidity of psychiatric disorders with BMI over 25 has been observed in both Qatar and UK. Yet, the lifestyles are different. Diet, exercise frequency and substance use have been shown to be significantly different between respondents of the two countries. Therefore, we conclude that this co-morbidity cannot be attributed to the same factors for all over the world, and further studies need to be done to understand the mechanisms involved in every situation. The results from this questionnaire address the necessity of developing more precision medicine approaches that consider the different lifestyles in population. The global problem of growing waistlines and psychiatric disorders can be better addressed by targeting a population’s specific needs to facilitate behavioral changes required to improve physical and mental health. We are proposing the idea that lifestyle factors can be involved in different manners and with different weights in resulting with such co-morbidity (Figure 2). In Qatar, the cause could be first attributed to a lack of exercise, and a diet which has shifted from the more traditional Arabic cuisine to that of the many cultures it now houses, including the high fat, processed Western diet. Although no difference in use of tobacco was found, it would be interesting to investigate on the role of Shisha smoking in this co-morbidity. For UK residents, the consumption of alcohol is more worrying, as many do not factor into their diet, but contributes 7 Kcal/g.

When addressing weight loss, the most effective way for an individual to maintain weight loss is to combine an exercise routine with a balanced diet and a change in overall lifestyle behaviors, which in turn will benefit overall quality of life and help to lessen the burden of psychiatric disorders. Most importantly, this survey allowed us to raise a concern that an important proportion of individuals seemed unaware of their BMI greater than 25 and/or of their psychiatric condition, and we would like to remind the importance of awareness as being the initial step to engage behavioral change.

## Data availability statement

The raw data supporting the conclusions of this article will be made available by the authors, without undue reservation.

## Ethics statement

The studies involving human participants were reviewed and approved by WCMQ-IRB. The patients/participants provided their written informed consent to participate in this study.

## Author contributions

GB conceived and designed the study and recruited participants. CG and GB developed the questionnaire and drafted the initial manuscript. GB, CG, and PS conducted data analysis. GB, PS, and EA made critical revisions of the manuscript for important intellectual content. All authors contributed to the article and approved the submitted version.

## Funding

The publication of this article was funded by Weill Cornell Medicine- Qatar Health Sciences library.

## Conflict of interest

The authors declare that the research was conducted in the absence of any commercial or financial relationships that could be construed as a potential conflict of interest.

## Publisher’s note

All claims expressed in this article are solely those of the authors and do not necessarily represent those of their affiliated organizations, or those of the publisher, the editors and the reviewers. Any product that may be evaluated in this article, or claim that may be made by its manufacturer, is not guaranteed or endorsed by the publisher.
