# Supplementary material for: Identifying lifestyle factors associated to co-morbidity of obesity and psychiatric disorders, a pilot study
Source: Front Public Health. 2023 May 3;11:1132994. doi: 10.3389/fpubh.2023.1132994 (PMC10188954; doi:10.3389/fpubh.2023.1132994)
Supplement: Supplementary file 1 [file Data_Sheet_1.PDF]

ASSESSING LIFESTYLE OF POPULATIONS AND RAISING AWARENESS ON ITS IMPACT ON HEALTH.

Link to quiz:
https://weillcornell.az1.qualtrics.com/jfe/form/SV_cLNbxSdMV4VjGaF

You are invited to participate in the study "Assessing lifestyle of populations and raising awareness on its impact on health".
This quiz will take you no longer than 15 minutes.
By submitting the quiz you are consenting that you are 18 years + and willing to participate in this study.
Participation is voluntary and your information will not be identified, if the survey is submitted we will not be able to delete your responses. All data will be analyzed confidentially. Therefore, if you do not want to participate, you should not submit the quiz.
ALL answers are completely anonymous.
After each question, you will be provided with a score on how healthy your lifestyle is compared to evidence-based and published guidelines.
Medical disclaimer: This quiz does not provide medical advice. It is intended for informational purposes only. It is not a substitute for professional medical advice, diagnosis or treatment.

Click the next button to get started

Question	Score	Feedback	Reference
**Anthropometric**			
Age<br>(if under 18 taken to end of quiz)	Not scored		
Height (cm)	Not scored		
Weight (kg)	Not scored		
Have you ever been diagnosed with any of the following medical conditions by a GP/physician: Diabetes type 1, Diabetes type 2, Cardiovascular disease, Anxiety, Obesity, Cancer (if selected prompted to name which type), Inflammatory bowel disease,	Not scored		

Rheumatoid arthritis, Autoimmune disease, Autism, Depression, Schizophrenia, Biopolar, Alzheimers, Parkinsons, Arthritis.			
Are you taking any prescribed medication for any condition selected in the previous question?	Not scored		
Gender:<br>Male, Female, non-binary/third gender	Not scored		
To which of the following ethnic groups do you belong?<br>Causcasian (white American, European, British, Turks, Australian)<br>Arab (Middle East)<br>North African<br>African/African American<br>Asian<br>South East Asian (Indian, Pakistan, Sri Lanka, Bangladesh)	Not scored		
What is your country of resisdence?<br>Qatar<br>Other	Not scored		
(Asked if the answer to country of residence is other)	Not scored		

Please tell us the country you reside in			
What is your highest level of education?<br>Primary school<br>Secondary school,<br>College<br>University<br>No Education	Not scored		
**NUTRITION**			
On average how many caffeinated drinks do you consume in a day (tea, coffee, soda, energy drinks)?	0=0<br>1=1<br>2=2<br>3=3	The FDA advises drinking no more than 4-5 coffees a day Good job, you are drinking under the recommended maximum amount of caffeine a day!	https://www.fda.gov/consumers/consumer-updates/spilling-beans-how-much-caffeine-too-much#:~:text=For%20healthy%20adults%2C%20the%20FDA,it%20(break%20it%20down).
	4+=4	The FDA advises drinking no more than 4-5 coffees a day. You may need to take a look at the amount of caffeine you are consuming!	
On average how much water do you drink in a day?<br><br>Under 1 litre<br>1-2 litres<br>Over 3 litres	Not scored		

On average how many servings of vegetables do you consume in a day?<br>1 serving= 1 cup raw leafy, about size of fist, 1/2 cup cooked, 1/2 cup of vegetable juice.	0=4<br>1=3<br>2=2<br>3=1	According to the American Heart Association based on a 2000 calorie diet, you should consume 4-5 servings of vegetables a day.<br>It seems you need to increase the number of vegetables you are eating, there is a link at the end of the quiz to recipes that can help you with this.	https://www.heart.org/en/healthy-living/healthy-eating/add-color/fruits-and-vegetables-serving-sizes
	4+=0	According to the American Heart Association based on a 2000 calorie diet, you should consume 4-5 servings of vegetables a day.<br>Good job, you are eating the recommended daily amount of vegetables!	
On average how many servings of fruit do you consume in a day?<br>1 serving= Size of baseball, 1/4 cup of dried fruit, 1/2 cup of fresh, frozen or canned, 1/2 cup of 100% fruit juice.	0=4<br>1=3<br>2=2<br>3=1	According to the American Heart Association based on a 2000 calorie diet, you should consume 4-5 servings of fruit a day.<br>It seems you need to increase the amount of fruit you are eating, there is a link to recipes at the end of the quiz to recipes that can help you with this.	https://www.heart.org/en/healthy-living/healthy-eating/add-color/fruits-and-vegetables-serving-sizes

	4+=0	According to the American Heart Association based on a 2000 calorie diet, you should consume 4-5 servings of fruit a day.<br>Good job, you are eating the recommended daily amount of fruit!	
On average how many servings of dairy do you consume in a day?<br>1 serving = 1 cup of low fat milk, 1 cup of low fat yogurt, 1 1/2 ounces of cheese.	0=4<br>1=3	According to the American Heart Association based on a 2000 calorie diet you should consume 2-3 servings of dairy a day.<br>It seems you are not consuming enough dairy. Dairy is important to help maintain bone density and reduces the risk of fractures.	https://www.heart.org/en/healthy-living/healthy-eating/eat-smart/nutrition-basics/dairy-products-milk-yogurt-and-cheese<br><br>https://www.health.harvard.edu/blog/dairy-health-food-or-health-risk-2019012515849
	2=0<br>3=0	According to the American Heart Association based on a 2000 calorie diet you should consume 2-3 servings of dairy a day.<br><br>Good job, you are consuming the recommended daily amount of dairy!	

	4+=2	According to the American Heart Association based on a 2000 calorie diet you should consume 2-3 servings of dairy a day.<br><br>It seems you are consuming a lot of dairy, dairy can be high in saturated fats.	
On average how many servings of lean meats, poultry and seafood do you consume in a day?<br>1 serving= 3 ounces of cooked meat, size of a computer mouse.	0=4	According to the American Heart Association based on a 2000 calorie diet you should consume less than 6 ounces of lean meats, poultry and seafood a day. You are not eating the recommended level of lean meats, poultry and seafood. There is a link at the end of the quiz to recipes that can help you with this.	https://www.heart.org/en/healthy-living/healthy-eating/eat-smart/nutrition-basics/meat-poultry-and-fish-picking-healthy-proteins
	1=1<br>2=0	According to the American Heart Association based on a 2000 calorie diet you should consume less than 6 ounces of lean meats, poultry and seafood a day.<br>Well done you are consuming the recommended amount of lean meats, poultry and seafood!	

	3=2<br>4+=3	According to the American Heart Association based on a 2000 calorie diet you should consume less than 6 ounces of lean meats, poultry and seafood a day.<br>Be careful, you seem to be eating a lot of meat. High consumption of meat can raise cholesterol levels.	
On average how many meals do you eat out/ take away in a week?	0=0<br>1=1<br>2=2<br>3=3<br>4+=4	The Scientific Report of the 2015 Dietary Guidelines Advisory Committee advises that you should aim to keep calorie intake to less than 10% from saturated fats and keep trans fats consumption as low as possible.	Loma Linda University, school of Medicine (Question)<br>https://health.gov/sites/default/files/2019-09/Scientific-Report-of-the-2015-Dietary-Guidelines-Advisory-Committee.pdf<br>(feedback)
Which of the following foods do you eat the most? Drag into place<br>(1=most eaten, 8= least eaten)<br>Red meat<br>Poultry<br>Pasta<br>Rice<br>Cheese<br>Pizza/Sandwich/Hamburger<br>Vegetables	Not scored		

Pastirs/ Sweets			
Do you have any food allergies or sensitivities?<br>Yes<br>No	Not scored		Loma Linda University, school of Medicine
Are you currently following any particular diet or nutrition plan?<br>Yes<br>No	Not scored		Loma Linda University, school of Medicine
Do you eat organic products?<br>All the products I eat are organic<br>I always eat organic products<br>Often<br>Sometimes<br>Never	Not scored		
(Only asked is Qatar is country of residence)<br>Please rank the following vegetables from the one you eat the most to the one you eat the least.<br>(1=most eaten, 7= least eaten<br>Potatoes, Tomatoes, Lettuce, Carrots, Cauliflower, Cucumber, eggplant/aubergine	Not scored		
Do you wash your vegetables<br>Yes<br>No	Not scored		
(If yes is selected to previous question)	Not scored		

What do you wash your vegetables in?<br>Water<br>Bleach<br>Vinegar			
Do you regularly consume sources of probiotics (Kimchi, yoghurt, kefir, kombucha, sauerkraut, pickles, miso, tempeh, sourdough bread and some cheeses)<br>Yes<br>No	Not scored		
**PHYSICAL ACTIVITY**		Any bodily movement produced by the contraction of skeletal muscle that increases energy expenditure above a basal level.	**https://health.gov/sites/default/files/2019-09/Physical_Activity_Guidelines_2nd_edition.pdf#page=56**
Medical disclaimer: This quiz does not provide medical advice. It is intended for informational purposes only. According to the American College of Sport Medicine guidelines and the American College of Lifestyle Medicine, if you have symptoms of metabolic disease, cardiovascular disease or renal disease, or if you have been diagnosed with one of			

these diseases, you must obtain medical clearance before engaging in any exercise.			
In an average week, how many days do you do exercise?	0=3	Information from the U.S. department of health and human services states the more you move the more health benefits you will gain, this has to be balanced with allowing your body to recover.<br>Obesity, type 2 diabetes, cardiovascular disease, anxiety & depression, osteoporosis, hypertension and some cancers such as breast and colon cancers are all at an increased risk from sedentary behaviour.	https://health.gov/our-work/physical-activity/current-guidelines/scientific-report<br><br>https://www.cdc.gov/physicalactivity/basics/adults/index.htm#:~:text=Physical%20activities%20to%20strengthen%20your,abdomen%2C%20shoulders%2C%20and%20arms.
	1-3=2	Based on information from the U.S. department of health and human services you are making a good effort by getting your body moving, see if you can increase the days you are active.	

	4-6=1<br>7+=0	Congratulations, based on information from the U.S. Department of health and human services you regularly undertake physical activity which has great health benefits.	
How intense is the exercise you do?	Low intensity=3	Based on information from the Canadian Academy of Sports Nutrition you aren't quite at the recommended intensity level.	https://www.caasn.com/sports-nutrition/energy-map/metabolism-of-fat-during-exercise.html
	Medium intensity=2<br>Vigorous intensity=1	Well done, based on information from the Canadian Academy of Sports Nutrition your exercise intensity matches or exceeds the guidelines.	
During an average week, how many minutes/hours approximately do you do exercise?	60 minutes or less=4<br>1-3 hours=3<br>4-7 hours=3<br>8 hours+=1	Based on information from the U.S. department of health and human services it is recommended that you do a minimum of 150 minutes of moderate exercise a week or 75 minutes of vigorous exercise a week.	https://health.gov/our-work/physical-activity/current-guidelines/scientific-report

Tick the types of Physical activity you do and how many days a week you do it.<br>Lifestyle exercise. (Taking the stairs, walking the dog etc)<br>Strength and resistance training. (Free weights, powerlifting etc)<br>Cardiovascular/ endurance. (Running, swimming, cycling etc)<br>Flexibility/balance. (static/ dynamic stretching, yoga etc)	0=3, 1-3=2, 4-6=1, 7=0<br><br>0=3, 1-3=0, 4-6=1, 7=2<br><br>0=3, 1-3=0, 4-6=1, 7=2<br><br>0=3, 1-3=0, 4-6=1, 7=2	Recommendations from the UK government website show that you should do<br><br>- 2-3 strength and resistance training sessions a week<br><br>- 2-3 flexibility and balance sessions a week<br><br>- 2 hours 30 minutes of moderate or 1 hour 15 minutes of vigorous cardiovascular exercise a week	https://health.gov/our-work/physical-activity/current-guidelines/scientific-report (question)<br>https://assets.publishing.service.gov.uk/government/uploads/system/uploads/attachment_data/file/829884/3-physical-activity-for-adults-and-older-adults.pdf (feedback)
		**Lower the score the closer to guidelines.**	
**Stress and emotional wellbeing**			
Depression scale-<br>1. Little interest or pleasure in doing things<br>2. Feeling down, depressed, or hopeless<br>3. Trouble falling or staying asleep, or sleeping too much<br>4. Feeling tired or having little energy<br>5. Poor appetite or overeating	Not at all =0<br>Several days =1<br>More than half the days =2<br>Nearly every day=3	According to the American Psychological Association:<br><br>1-4 Minimal depression<br>5-9 Mild depression<br>10-14 Moderate depression<br>15-19 Moderately severe depression<br>20-27 Severe depression	Questions from:<br>https://www.apa.org/pi/about/publications/caregivers/practice-settings/assessment/tools/patient-health

6. Feeling bad about yourself or that you are a failure or have let yourself or your family down<br>7. Trouble concentrating on things, such as reading the newspaper or watching television<br>8. Moving or speaking so slowly that other people could have noticed. Or the opposite being so figety or restless that you have been moving around a lot more than usual<br>9. Thoughts that you would be better off dead, or of hurting yourself			
Perceived stress scale.<br>In the last month, how often have you felt that you were unable to control important things in your life?<br>In the last month, how often have you felt confident about your ability to handle your personal problems?<br>In the last month, how often have you felt that things were going your way?	Questions 1+4<br>• 0=Never<br>• 1-Almost never<br>• 2=Sometimes<br>• 3=Fairly often<br>• 4=Very often<br><br>Questions 2+3<br>• 4=Never<br>• 3=Almost never<br>• 2=Sometimes<br>• 1=Fairly often	• Scores 0-4 low stress<br>• Scores 5-9 Moderate stress<br>• Scores 10-16 High perceived stress	American College of lifestyle medicine<br>Cohen, S., Kamarck, T., & Mermelstein, R. (1983). A global measure of perceived stress. Journal of Health and Social Behavior, 24, 385-396

In the last month, how often have you felt difficulties were piling up so high that you could not over come them?	• 0=Very often		
		The lower the scores the less likely you are to be stressed or feel depressed.	
**Sleep**			
Based on an average night, how many hours do you sleep?	Less than 4 hours=1<br>4-6 hours=2	According to the NHS you do not seem to be getting the recommended amount of sleep.<br>Benefits of sleep:<br>Improved memory and learning<br>Growth hormone released during deep sleep	https://www.nhs.uk/live-well/sleep-and-tiredness/how-to-get-to-sleep/#:~:text=Most%20adults%20need%20between%206,the%20same%20time%20every%20day.
	7-9 hours=3<br>10+ hours=3	Well done, according to the NHS you are achieving the recommended amount of sleep per night.<br>Benefits of sleep:<br>Improved memory and learning<br>Growth hormone released during deep sleep	
Do you use any electronic devices such as televisions,	Yes<br>No	If yes is selected:<br>According to the NHS electronic gadgets, lights and noise can	https://www.nhs.uk/live-well/sleep-and-tiredness/how-to-get-to-

computer consoles, laptops or mobile phones in the bedroom?		weaken the association between the bedroom and sleep.	sleep/#:~:text=Most%20adults%20need%20between%206,the%20same%20time%20every%20day<br>.<br>Lockley sw, Foster rg, sleep:a very short introduction. Vol 295oxford university press:2012
Do you feel like you get enough sleep?	Not scored		
**Substance use**			
Do you use any nicotine/tobacco products? (cigarettes, e-cigarettes/vaping, cigars, shisha)	Yes<br>No	If yes selected- The centres for disease control and prevention (CDC) show smoking increases the risk:<br>For coronary heart disease by 2 to 4 times<br>For stroke by 2 to 4 times<br>Of men developing lung cancer by 25 times<br>Of women developing lung cancer by 25.7 times	https://www.cdc.gov/tobacco/data_statistics/fact_sheets/health_effects/effects_cig_smoking/#reduced-risks
How many times a day do you use your chosen nicotine/tobacco product? (Question only available if answer yes to previous question)	10 or less<br>11-20<br>21+	Information from the CDC shows if you quit smoking, your risks for cancers of the mouth, throat, esophagus, and bladder drop by half within 5 years.	https://www.cdc.gov/tobacco/data_statistics/fact_sheets/health_effects/effects_cig_smoking/#reduced-risks
Do you drink alcohol?	Yes<br>No		

What type of alcohol do you mainly drink? (question only available if answer yes to previous question)	Beer<br>Wine<br>Spirits		
How many units of your chosen alcohol do you drink in an average week? (1 pint of beer = 2.3 units, 1 large wine glass= 2.3 units, 1 single spirit= 1 unit)	1-13<br>14+	The National Institute of Alcohol Abuse and Alcoholism states that men should consume no more than 4 drinks a day or more than 14 drinks in 7 days and women should drink no more than 3 drinks a day or no more than 7 drinks in any 7 days.	https://pubs.niaaa.nih.gov/publications/practitioner/cliniciansguide2005/guide.pdf
		• Tobacco: Premature aging, Respiratory infections and asthma. Heart disease, stroke, vascular disease<br>• Alcohol: Aggressive and violent behaviour, Reduced sexual performance, Digestive problems, ulcers, inflammation of the pancreas, high blood pressure	https://www.who.int/substance_abuse/activities/en/Draft_The_ASSIST_Guidelines.pdf?ua=1

Did you know….

<table>
<tr><td>Nutrition:</td><td></td></tr>
<tr><td>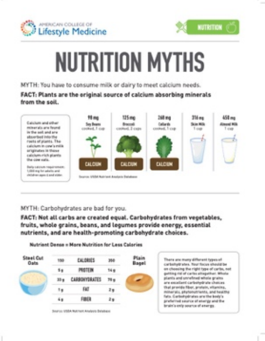</td><td>American College of Lifestyle Medicine</td></tr>
<tr><td>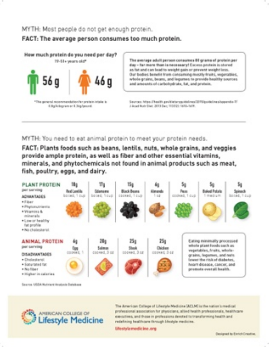</td><td>American College of Lifestyle Medicine</td></tr>
<tr><td>Physical Activity:</td><td></td></tr>
<tr><td>Every sweat session you do can help strengthen your immune function for about 24 hours.</td><td>https://www.acefitness.org/education-and-resources/lifestyle/exercise-library/</td></tr>
<tr><td>Emotional wellbeing:</td><td></td></tr>
</table>

Researchers at the University of Kansas published findings that smiling helps reduce the body's response to stress and lower heart rate in tense situations	https://www.psychologicalscience.org/news/releases/smiling-facilitates-stress-recovery.html
**Sleep:**	
People who sleep less, eat more. This is because of decreased levels of the hormone 'leptin', which regulates the appetite, and helps well-rested people control their cravings for food.	https://www.open.edu.au/advice/insights/10-fun-and-useful-facts-about-sleep
**Substance use:**	
Women have less alcohol dehydrogenase, an enzyme that breaks down alcohol before it reaches the bloodstream. As a result, one drink for a woman is roughly equivalent to two drinks for a man.	https://www.health.harvard.edu/womens-health/why-does-alcohol-affect-women-differently
Men who quit smoking by the age of 30 add 10 years to their life. People who kick the habit at 60 add 3 years to their life.	https://www.nhs.uk/live-well/quit-smoking/?tabname=smoking-facts

Table 1: Top consumed foods in Qatar and UK by all respondents

Qatar	UK
Poultry n=76	Poultry n=29
Vegetables n=58	Vegetables n=10
Rice n=56	Red meat n=8
Red meat n=31	Pasta n=5
Pasta n=15	Rice, pizza, sandwich, hamburger n=3
Total=236	Total =55

*Number of respondents from Qatar who answered this question in the questionnaire, n=236.
Number of respondents from the UK who answered this question in the questionnaire, n=55

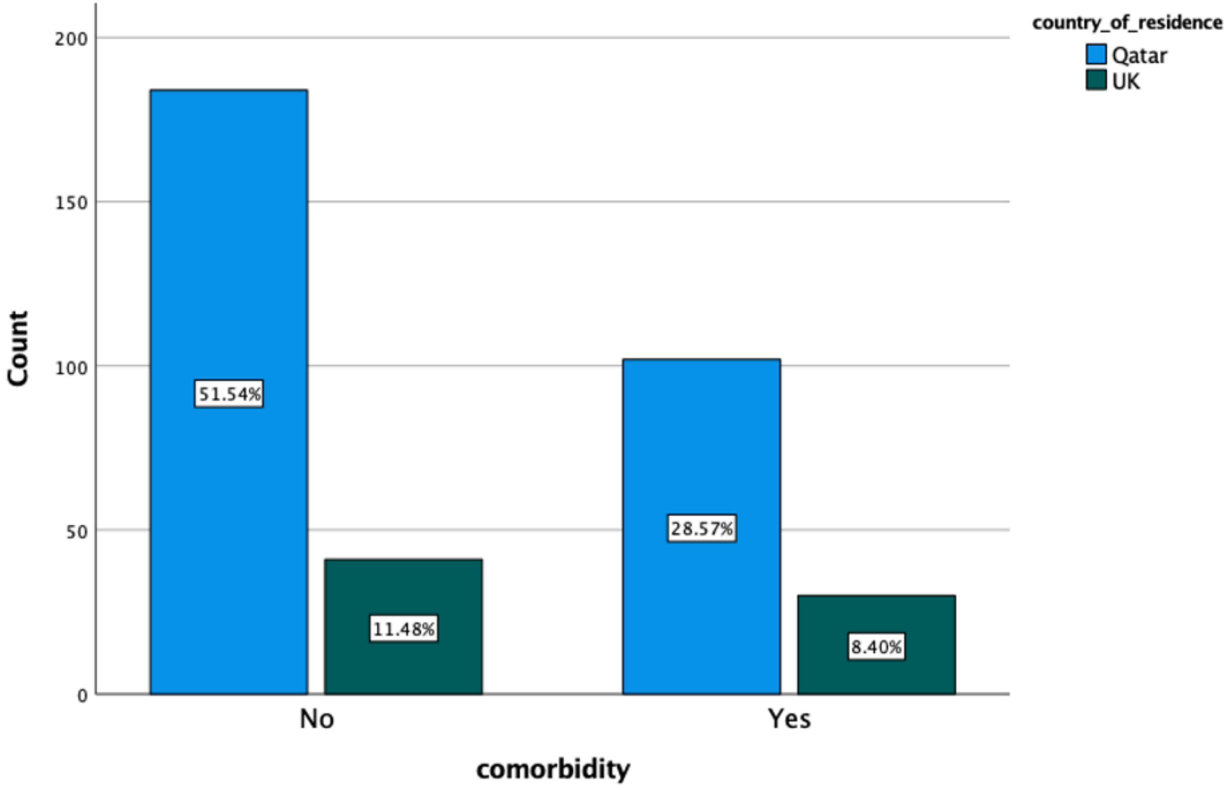
country_of_residence
Qatar
UK
Count
200
150
100
50
0
51.54%
11.48%
28.57%
8.40%
No
Yes
comorbidity

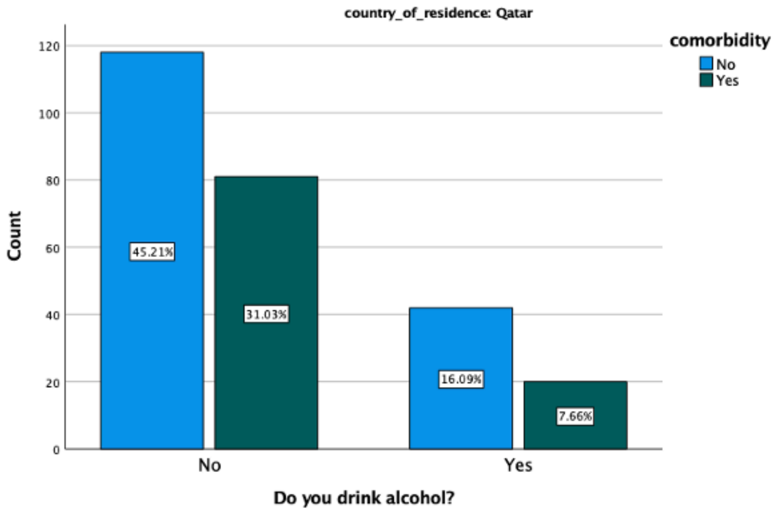

country_of_residence: Qatar
comorbidity
No
Yes
Count
120
100
80
60
40
20
0
45.21%
31.03%
16.09%
7.66%
No
Yes
Do you drink alcohol?

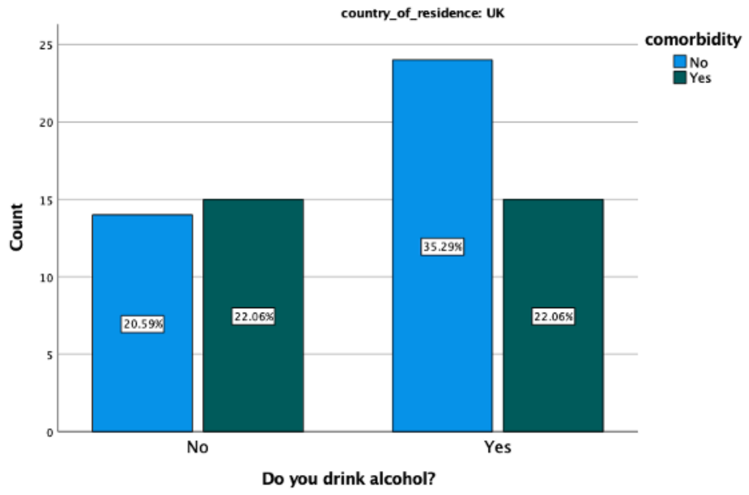

country_of_residence: UK
comorbidity
No
Yes
Count
25
20
15
10
5
0
20.59%
22.06%
35.29%
22.06%
No
Yes
Do you drink alcohol?

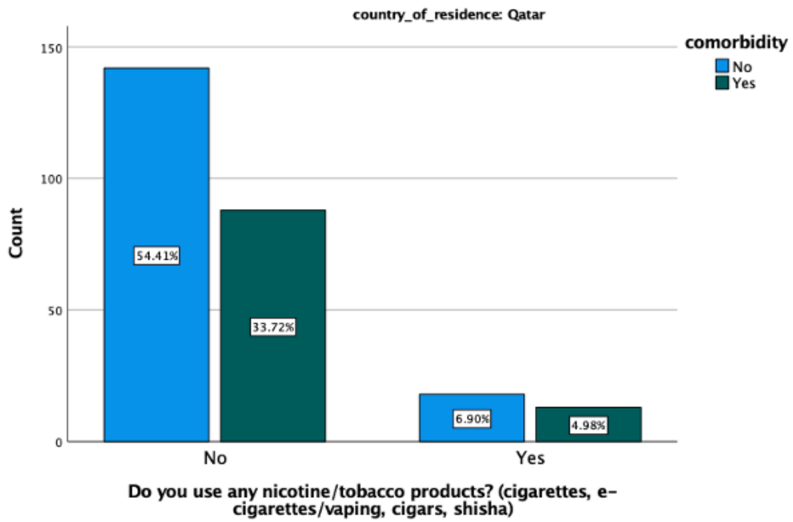
country_of_residence: Qatar
comorbidity
No
Yes
Count
150
100
50
0
54.41%
33.72%
6.90%
4.98%
No
Yes
Do you use any nicotine/tobacco products? (cigarettes, e-cigarettes/vaping, cigars, shisha)

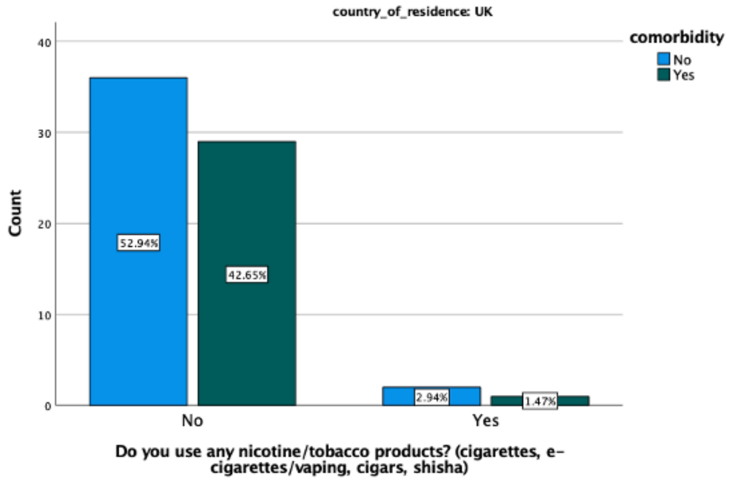

country_of_residence: UK
comorbidity
No
Yes
Count
40
30
20
10
0
52.94%
42.65%
2.94%
1.47%
No
Yes
Do you use any nicotine/tobacco products? (cigarettes, e-cigarettes/vaping, cigars, shisha)

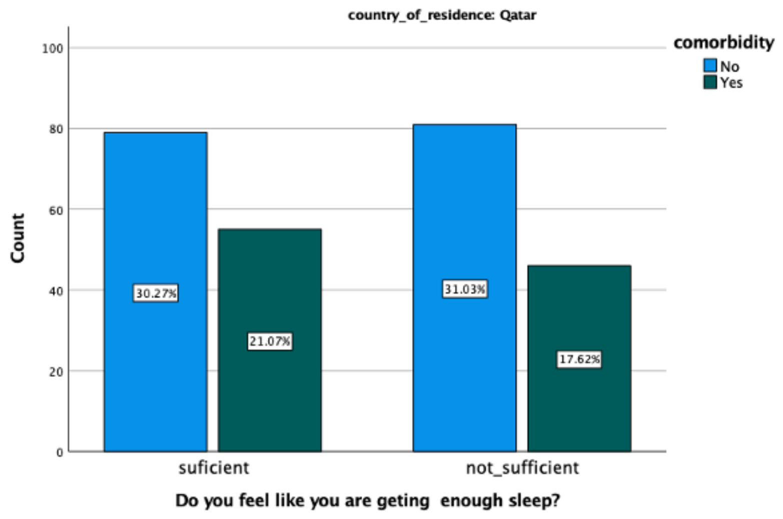
country_of_residence: Qatar
comorbidity
No
Yes
Count
100
80
60
40
20
0
30.27%
21.07%
31.03%
17.62%
suficient
not_sufficient
Do you feel like you are geting enough sleep?

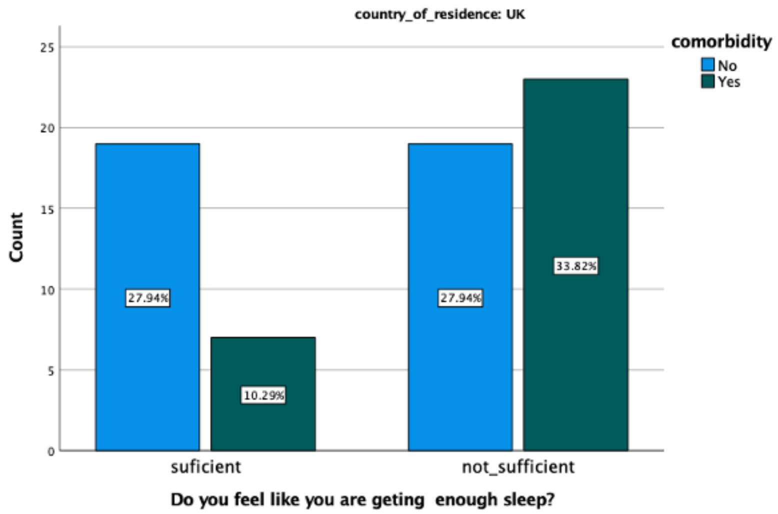

country_of_residence: UK
comorbidity
No
Yes
Count
25
20
15
10
5
0
27.94%
10.29%
27.94%
33.82%
suficient
not_sufficient
Do you feel like you are geting enough sleep?

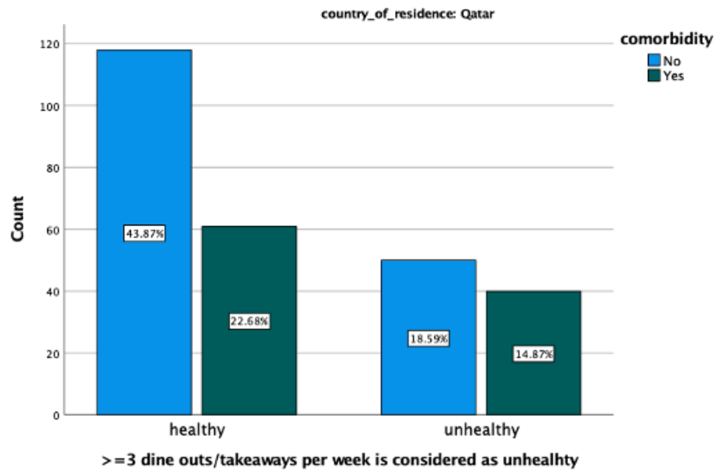
country_of_residence: Qatar
comorbidity
No
Yes
Count
120
100
80
60
40
20
0
43.87%
22.68%
18.59%
14.87%
healthy
unhealthy
>=3 dine outs/takeaways per week is considered as unhealhty

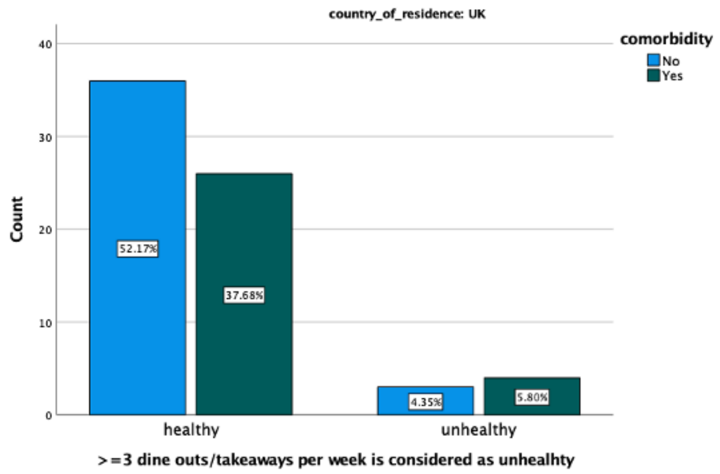

country_of_residence: UK
comorbidity
No
Yes
Count
40
30
20
10
0
52.17%
37.68%
4.35%
5.80%
healthy
unhealthy
>=3 dine outs/takeaways per week is considered as unhealhty

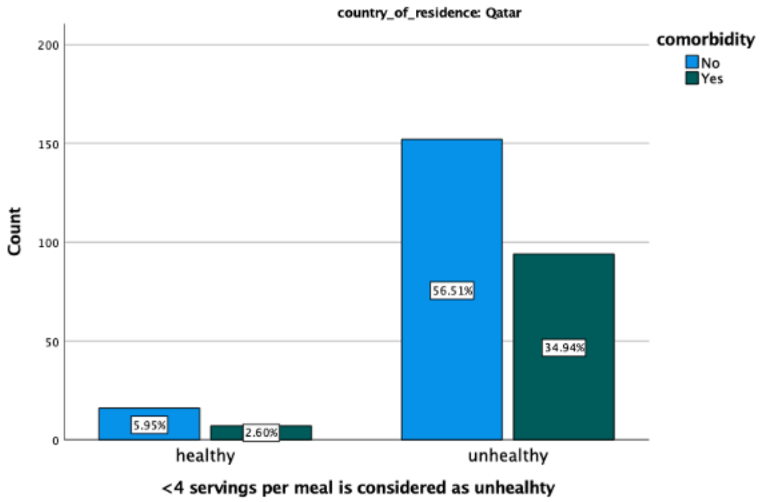

country_of_residence: Qatar
comorbidity
No
Yes
Count
200
150
100
50
0
5.95%
2.60%
56.51%
34.94%
healthy
unhealthy
<4 servings per meal is considered as unhealhty

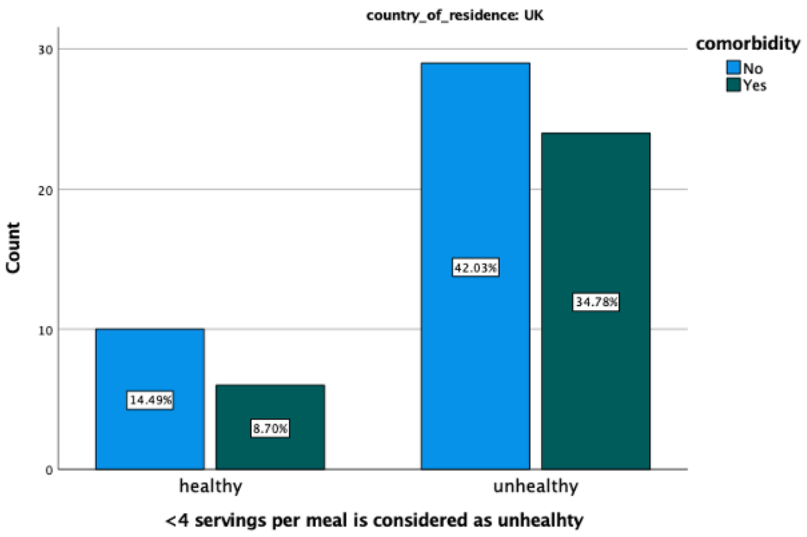

country_of_residence: UK
comorbidity
No
Yes
Count
30
20
10
0
14.49%
8.70%
42.03%
34.78%
healthy
unhealthy
<4 servings per meal is considered as unhealhty

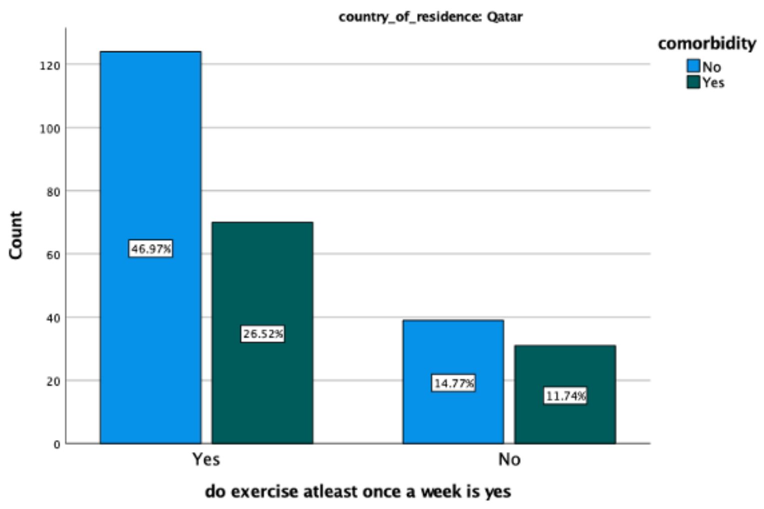

country_of_residence: Qatar
comorbidity
No
Yes
Count
120
100
80
60
40
20
0
46.97%
26.52%
14.77%
11.74%
Yes
No
do exercise atleast once a week is yes

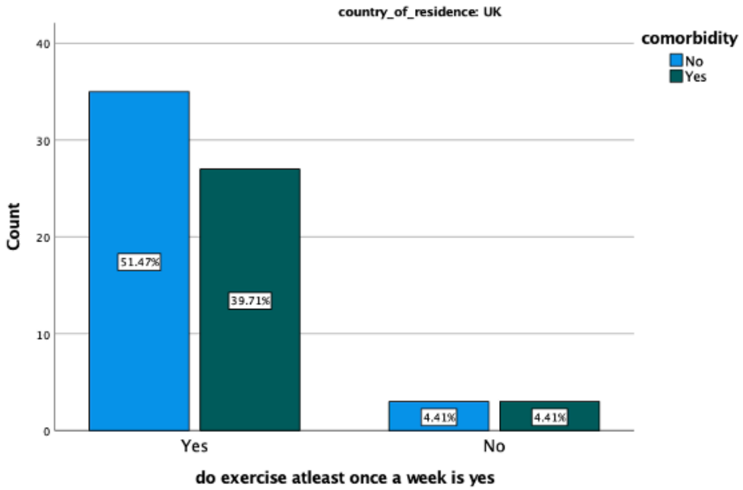

country_of_residence: UK
comorbidity
No
Yes
Count
40
30
20
10
0
51.47%
39.71%
4.41%
4.41%
Yes
No
do exercise atleast once a week is yes

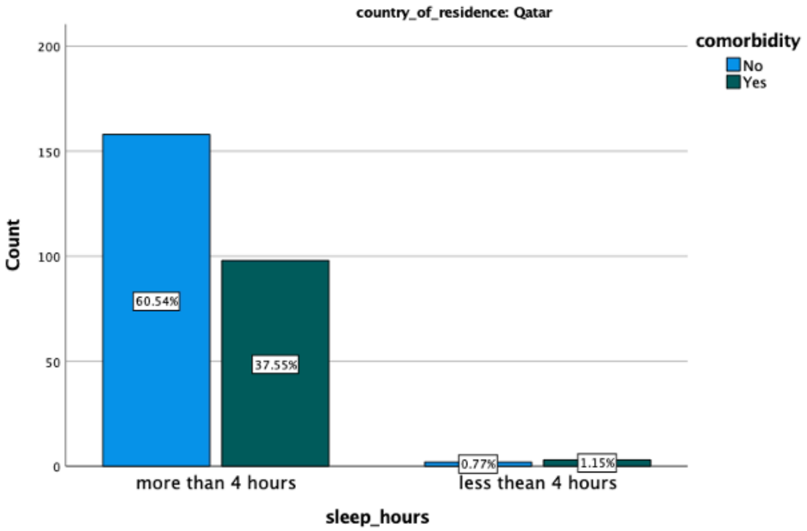
country_of_residence: Qatar
comorbidity
No
Yes
Count
200
150
100
50
0
60.54%
37.55%
0.77%
1.15%
more than 4 hours
less thean 4 hours
sleep_hours

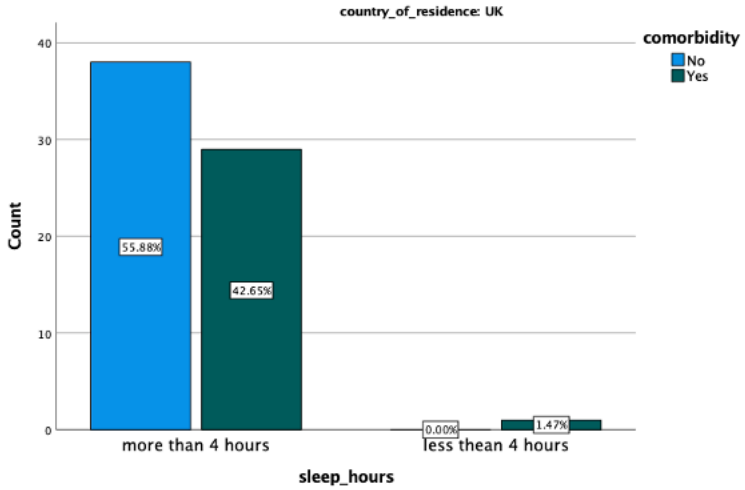

country_of_residence: UK
comorbidity
No
Yes
Count
40
30
20
10
0
55.88%
42.65%
0.00%
1.47%
more than 4 hours
less thean 4 hours
sleep_hours
