# Supplementary material for: Identifying lifestyle factors associated to co-morbidity of obesity and psychiatric disorders, a pilot study
Source: Front Public Health. 2023 May 3;11:1132994. doi: 10.3389/fpubh.2023.1132994 (PMC10188954; doi:10.3389/fpubh.2023.1132994)
Supplement: Supplementary file 2 [file Data_Sheet_2.docx]

**Table 1: Case processing summary Qatar**

|  | | **N** | **Marginal Percentage** |
| --- | --- | --- | --- |
| Comorbid | No | 160 | 61.3% |
|  | Yes | 101 | 38.7% |
| Alcohol consumption | No | 199 | 76.2% |
|  | Yes | 62 | 23.8% |
| Tobacco consumption | No | 230 | 88.1% |
|  | Yes | 31 | 11.9% |
| Perception of having enough sleep | Yes | 134 | 51.3% |
|  | No | 127 | 48.7% |
| Takeaways/ dining out in a week | <3 per week | 172 | 65.9% |
|  | ≥3 per week | 89 | 34.1% |
| Daily vegetable serving | ≥4 servings daily | 23 | 8.8% |
|  | <4 servings daily | 238 | 91.2% |
| Exercise at least once a week | Yes | 193 | 73.9% |
|  | No | 68 | 26.1% |
| Sleep hours | ≥4 hours or more | 256 | 98.1% |
|  | <4 hours | 5 | 1.9% |
| Valid | | 261 | 100.0% |
| Missing | | 25 |  |
| Total | | 286 |  |

|  |
| --- |
| **Table 2: Case processing Summary UK**   \|  \| \| **N** \| **Marginal Percentage** \| \| --- \| --- \| --- \| --- \| \| Comorbid \| No \| 38 \| 55.9% \| \| Yes \| 30 \| 44.1% \| \| Alcohol consumption \| No \| 29 \| 42.6% \| \| Yes \| 39 \| 57.4% \| \| Tobacco consumption \| No \| 65 \| 95.6% \| \| Yes \| 3 \| 4.4% \| \| Perception of having enough sleep \| Yes \| 26 \| 38.2% \| \| No \| 42 \| 61.8% \| \| Takeaways/ dining out in a week \| <3 per week \| 61 \| 89.7% \| \| ≥3 per week \| 7 \| 10.3% \| \| Daily vegetable serving \| ≥4 servings daily \| 16 \| 23.5% \| \| <4 servings daily \| 52 \| 76.5% \| \| Exercise at least once a week \| Yes \| 62 \| 91.2% \| \| No \| 6 \| 8.8% \| \| Sleep hours \| ≥4 hours or more \| 67 \| 98.5% \| \| <4 hours \| 1 \| 1.5% \| \| Valid \| \| 68 \| 100.0% \| \| Missing \| \| 3 \|  \| \| Total \| \| 71 \|  \| |

**Table 3: Model fitting Qatar**

| **Model** | **Model Fitting Criteria** | | | **Likelihood Ratio Tests** | | |
| --- | --- | --- | --- | --- | --- | --- |
|  | **AIC** | **BIC** | **-2 Log Likelihood** | **Chi-Square** | **df** | **Sig.** |
| **Intercept Only** | 115.939 | 119.504 | 113.939 |  |  |  |
| **Final** | 122.508 | 151.024 | 106.508 | 7.431 | 7 | 0.385 |

|  |
| --- |

**Table 4: Model fitting UK**

| **Model** | **Model Fitting Criteria** | | | **Likelihood Ratio Tests** | | |
| --- | --- | --- | --- | --- | --- | --- |
|  | **AIC** | **BIC** | **-2 Log Likelihood** | **Chi-Square** | **df** | **Sig.** |
| **Intercept Only** | 53.805 | 56.025 | 51.805 |  |  |  |
| **Final** | 59.031 | 76.787 | 43.031 | 8.775 | 7 | 0.269 |

| **Table 5: Goodness-of-Fit Qatar**   \|  \| **Chi-Square** \| **df** \| **Sig.** \| \| --- \| --- \| --- \| --- \| \| **Pearson** \| 46.574 \| 34 \| 0.074 \| \| **Deviance** \| 55.313 \| 34 \| 0.012 \| |
| --- | --- | --- | --- | --- | --- | --- | --- | --- | --- | --- | --- | --- |

**Table 6: Goodness-of-Fit UK**

|  | **Chi-Square** | **df** | **Sig.** |
| --- | --- | --- | --- |
| **Pearson** | 24.295 | 12 | 0.019 |
| **Deviance** | 30.371 | 12 | 0.002 |

**Table 7: Variation in the outcome for Qatar explained by the model**

| **Pseudo R-Square** | |
| --- | --- |
| **Cox and Snell** | 0.028 |
| **Nagelkerke** | 0.038 |
| **McFadden** | 0.021 |

|  |
| --- |

**Table 8: Variation in the outcome for UK explained by the model**

| **Pseudo R-Square** | |
| --- | --- |
| **Cox and Snell** | 0.121 |
| **Nagelkerke** | 0.162 |
| **McFadden** | 0.094 |

**Table 9: Likelihood ratio test Qatar**

| **Effect** | **Model Fitting Criteria** | | **Likelihood Ratio Tests** | | |  |
| --- | --- | --- | --- | --- | --- | --- |
|  | **-2 Log Likelihood of Reduced Model** | **Chi-Square** | | **df** | **Sig.** | |
| Alcohol consumption | 107.417 | 0.909 | | 1 | 0.340 | |
| Tobacco consumption | 106.775 | 0.267 | | 1 | 0.606 | |
| Perception of having enough sleep | 107.920 | 1.411 | | 1 | 0.235 | |
| Takeaways/ dining out in a week | 107.627 | 1.119 | | 1 | 0.290 | |
| Daily vegetable serving | 107.006 | 0.498 | | 1 | 0.480 | |
| Exercise frequency | 107.815 | 1.306 | | 1 | 0.253 | |
| Sleep hours | 107.947 | 1.439 | | 1 | 0.230 | |

**Table 10: Likelihood ratio test UK**

| **Effect** | **Model Fitting Criteria** | | **Likelihood Ratio Tests** | | |  |
| --- | --- | --- | --- | --- | --- | --- |
|  | **-2 Log Likelihood of Reduced Model** | **Chi-Square** | | **df** | **Sig.** | |
| Alcohol consumption | 44.694 | 1.663 | | 1 | 0.197 | |
| Tobacco consumption | 43.031 | 0.000 | | 1 | 0.986 | |
| Perception of having enough sleep | 47.921 | 4.891 | | 1 | 0.027 | |
| Takeaways/ dining out in a week | 43.091 | 0.060 | | 1 | 0.807 | |
| Daily vegetable serving | 43.653 | 0.622 | | 1 | 0.430 | |
| Exercise frequency | 43.050 | 0.019 | | 1 | 0.890 | |
| Sleep hours | 44.162 | 1.131 | | 1 | 0.288 | |

**Table 11:** **Multivariate Logistic Regression Results for the combined sample- Predictors of comorbidity variable in Qatar and UK**

| **Parameter Estimates** | | | | | | | | | |
| --- | --- | --- | --- | --- | --- | --- | --- | --- | --- |
| **Characteristics (ref: no)** | | **B** | **Std. Error** | **Wald** | **df** | **Sig.** | **Exp(B)** | **95% Confidence Interval for Exp(B)** | |
|  |  |  |  |  |  |  |  | **Lower Bound** | **Upper Bound** |
|  | Alcohol consumption (ref: No) | -0.374 | 0.269 | 1.935 | 1 | 0.164 | 0.688 | 0.406 | 1.165 |
|  | Tobacco consumption (ref: No) | 0.162 | 0.380 | 0.183 | 1 | 0.669 | 1.176 | 0.559 | 2.475 |
|  | Sleep perception (ref: Yes, to the question, do you feel you get enough sleep?) | 0.032 | 0.233 | 0.019 | 1 | 0.889 | 1.033 | 0.655 | 1.630 |
|  | Dine out/take away (ref: <3 per week) | 0.333 | 0.261 | 1.627 | 1 | 0.202 | 1.395 | 0.837 | 2.326 |
|  | Daily Vegetable Servings (ref: ≥4 servings daily) | 0.293 | 0.373 | 0.616 | 1 | 0.433 | 1.340 | 0.645 | 2.787 |
|  | Exercise frequency (ref: Yes) | 0.228 | 0.285 | 0.639 | 1 | 0.424 | 1.256 | 0.718 | 2.196 |
|  | Country (ref: Qatar) | 0.525 | 0.308 | 2.907 | 1 | 0.088 | 1.691 | 0.924 | 3.093 |

**Table 12:** **Multivariate Logistic Regression Results- Predictors of comorbidity variable in Qatar**

| **Parameter Estimates^a^** | | | | | | | | | |  |
| --- | --- | --- | --- | --- | --- | --- | --- | --- | --- | --- |
| **Characteristics** | | **B** | **Std. Error** | **Wald** | **df** | **Sig.** | **Exp(B)** | **95% Confidence Interval for Exp(B)** | |  |
|  |  |  |  |  |  |  |  | **Lower Bound** | **Upper Bound** |  |
|  | Alcohol consumption (ref: No) | -0.303 | 0.320 | 0.894 | 1 | 0.344 | 0.739 | 0.394 | 1.384 |  |
|  | Tobacco consumption (ref: No) | 0.208 | 0.402 | 0.269 | 1 | 0.604 | 1.232 | 0.560 | 2.708 |  |
|  | Sleep perception (ref: Yes, to the question, do you feel you get enough sleep?) | -0.317 | 0.268 | 1.403 | 1 | 0.236 | 0.728 | 0.431 | 1.231 |  |
|  | Dine out/take away (ref: <3 per week) | 0.294 | 0.277 | 1.124 | 1 | 0.289 | 1.341 | 0.779 | 2.308 |  |
|  | Daily Vegetable Servings (ref: ≥4 servings daily) | 0.337 | 0.485 | 0.483 | 1 | 0.487 | 1.401 | 0.541 | 3.624 |  |
|  | Exercise frequency (ref: Yes) | 0.348 | 0.304 | 1.312 | 1 | 0.252 | 1.417 | 0.781 | 2.571 |  |
| a. country of residence = Qatar | | | | | | | | | | |
|  | | | | | | | | | | |

**Table 13: Multivariate Logistic Regression Results- Predictors of comorbidity variable in UK**

| **Parameter Estimates^a^** | | | | | | | | | | |
| --- | --- | --- | --- | --- | --- | --- | --- | --- | --- | --- |
| **Characteristics** | | | **B** | **Std. Error** | **Wald** | **df** | **Sig.** | **Exp(B)** | **95% Confidence Interval for Exp(B)** | |
|  |  |  |  |  |  |  |  |  | **Lower Bound** | **Upper Bound** |
|  | | Alcohol consumption (ref: No) | -0.704 | 0.553 | 1.623 | 1 | 0.203 | 0.494 | 0.167 | 1.461 |
|  |  | Tobacco consumption (ref: No) | 0.021 | 1.338 | 0.000 | 1 | 0.987 | 1.021 | 0.074 | 14.071 |
|  |  | Sleep perception (ref: Yes, to the question, do you feel you get enough sleep?) | 1.228 | 0.576 | 4.553 | 1 | 0.033 | 3.415 | 1.105 | 10.552 |
|  |  | Dine out/take away (ref: <3 per week) | 0.213 | 0.872 | 0.059 | 1 | 0.807 | 1.237 | 0.224 | 6.832 |
|  |  | Daily Vegetable Servings (ref: ≥4 servings daily) | 0.512 | 0.657 | 0.607 | 1 | 0.436 | 1.668 | 0.460 | 6.044 |
|  |  | Exercise frequency (ref: Yes) | -0.133 | 0.968 | 0.019 | 1 | 0.891 | 0.875 | 0.131 | 5.842 |
| a. country of residence = UK | | | | | | | | | | |
|  | | | | | | | | | | |

**Table 14: Top consumed foods in Qatar and UK by all respondents**

| **Qatar** | **UK** |
| --- | --- |
| Poultry= 76 | Poultry= 29 |
| Vegetables= 58 | Vegetables= 10 |
| Rice= 56 | Red meat= 8 |
| Red meat= 31 | Pasta= 5 |
| Pasta= 15 | Rice, pizza, sandwich, hamburger= 3 |
| Total= 236 | Total= 55 |

*Number of respondents from Qatar who answered this question in the questionnaire n=236. Number of respondents from UK who answered this question in the questionnaire n=55.
